# When Yield Prediction Does Not Yield Prediction: An Overview of the Current Challenges

**DOI:** 10.1021/acs.jcim.3c01524

**Published:** 2023-12-20

**Authors:** Varvara Voinarovska, Mikhail Kabeshov, Dmytro Dudenko, Samuel Genheden, Igor V. Tetko

**Affiliations:** † Molecular AI, Discovery Sciences R&D, AstraZeneca, 431 83 Gothenburg, Sweden; ‡ TUM Graduate School, Faculty of Chemistry, Technical University of Munich, 85748 Garching, Germany; § Enamine Ltd., 78 Chervonotkatska str., 02094 Kyiv, Ukraine; ∥ Molecular Targets and Therapeutics Center, Helmholtz Munich − Deutsches Forschungszentrum für Gesundheit und Umwelt (GmbH), Institute of Structural Biology, 85764 Neuherberg, Germany

## Abstract

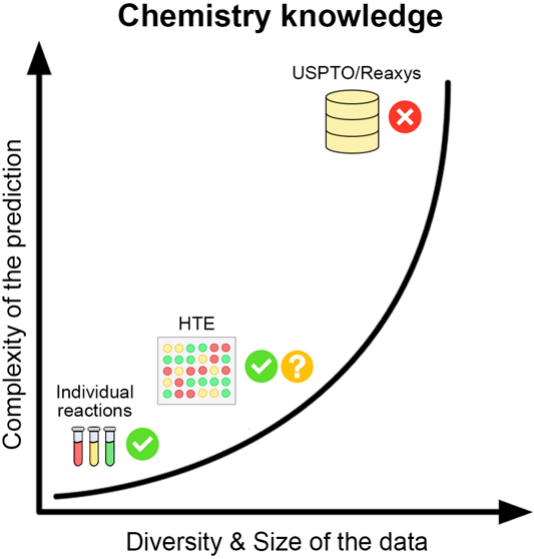

Machine Learning (ML) techniques face significant challenges when predicting advanced chemical properties, such as yield, feasibility of chemical synthesis, and optimal reaction conditions. These challenges stem from the high-dimensional nature of the prediction task and the myriad essential variables involved, ranging from reactants and reagents to catalysts, temperature, and purification processes. Successfully developing a reliable predictive model not only holds the potential for optimizing high-throughput experiments but can also elevate existing retrosynthetic predictive approaches and bolster a plethora of applications within the field. In this review, we systematically evaluate the efficacy of current ML methodologies in chemoinformatics, shedding light on their milestones and inherent limitations. Additionally, a detailed examination of a representative case study provides insights into the prevailing issues related to data availability and transferability in the discipline.

## 1 Introduction

Recent advancements in Machine Learning (ML) for chemistry have established these techniques as invaluable tools for predicting a wide range of properties associated with chemical reactions. Such tools typically fall under the umbrella of computer-assisted synthesis planning and include many different tools and models that can help chemists with several tasks. Retrosynthesis models suggest how to break a compound, either as a single-step prediction or multistep prediction, which provides a sequence of steps for how to synthesize a compound from simpler starting material.^1−3^ Furthermore, there are a range of product prediction models, or forward models that predict what the product of two or more reactants will be,^4,5^ or can provide guidance on regioselectivity issues.^6,7^ There are also condition or reagent models suggesting suitable catalysts, solvents, temperatures, etc.^8,9^ Finally, there are yield or reactivity models estimating the success of a reaction, which is the topic of this perspective and will be reviewed below. Although many encouraging studies have been reported, ML models for chemistry are not without critique.^10,11^ Furthermore, while many studies emphasize general reaction properties, such as yield prediction in regression and classification tasks, properties tied to physical chemistry, such as reaction rates and activation energies, have received less attention.

Reaction yield prediction holds particular significance in organic synthesis, especially within drug discovery and pharmaceutical development, where intricate multistep processes are routine. Any decrease in yield in a single step can drastically influence the overall success of the synthesis. Thus, crafting models that can predict yields for diverse pharmaceutically relevant reactions is crucial. Such predictive models offer myriad benefits, from trimming synthesis costs making drugs more affordable to curtailing the emergence of unwanted byproducts enhancing synthesis sustainability.

Historically, predicting reaction yields has been a challenging endeavor. In the 1940s, the Hammett equation emerged,^12^ a significant achievement in physical organic chemistry that linked reactivity and chemical structures. Moving to the 1980s, chemists started using basic methods to predict the properties of small organic molecules, and the first application of Neural Networks for Structure–Activity Relationship was introduced in 1992.^13^ The 2000s brought successes in QSAR (Quantitative Structure–Activity Relationship) using Random Forest and Support Vector Machines.^14−16^

From the late 1980s to the early 2010s, classical Machine Learning (ML) models started mimicking chemists’ rules for predicting physical properties and reaction outcomes, as described in a review by Williams et al.^17^ However, limited computational capabilities hindered advanced approaches. Yet, by the mid-2010s, advancements in microelectronics spurred the rise of sophisticated ML techniques. During this resurgence, Emami et al. achieved significant progress in 2015 by using thermodynamics calculations on a small set of compounds to achieve notable correlations.^18^ Later, Raccuglia et al. employed a support vector machine-based decision tree to predict reaction success.^19^ The public release of over a million reactions systematically extracted from patents in 2016^20^ drove further advancements, leading to more intricate models rooted in cutting-edge Deep Learning methods.^2,21,22^

To provide a comprehensive view of the present challenges in yield prediction, this work focuses on two key aspects: data and modeling. These aspects encompass the core of the current challenge. We also provide a section with our analysis of current approaches and challenges in modeling processing.

## 2 Data

The Data section is designed to provide an overview, spanning from the practical aspects of organic chemistry experimentation and data recording to the subsequent chemoinformatic modeling of reactions using these recorded representations. This structure takes us from the tangible, real-life processes to the digital domain, culminating in an exploration of the challenges encountered from both perspectives.

### 2.1 Experimental Methods to Generate Reaction Data

While an enormous amount of reaction data is already available, it is important to highlight a few exemplary, promising experimental approaches that facilitate high-quality reaction data generation in the modern Artificial Intelligence (AI)-driven era.

One of the key concepts developed in recent years is the automation of organic synthesis.^23^ This includes advances in automatic solid and liquid handling, precise dispensing, automatic compound purification using catch-and-release techniques, and the autonomous control of reaction parameters such as temperature, pressure, homogeneity, and color. Implementing reaction automation has increased the throughput of compound synthesis and reaction reproducibility by eliminating errors and mishandling from human interaction.

By combining automated synthesis and purification, researchers could generate 14 classes of organic compounds using the Suzuki-Miyaura cross-coupling reaction while recording high-quality reaction data.^24^

Further, increasing reaction data generation throughput can also be achieved by lowering the scale of individual experiments. This was exemplified in a study where more than 1500 Buchwald-Hartwig experiments were performed in less than a day using as little as 0.2 mg of starting material per reaction.^25^ However, it is crucial to note that the reaction data generated by this method can only be used for predicting reaction feasibility and rough yield estimation as no isolated yield information can be obtained.

Continuous flow chemistry methods are gaining popularity in the synthesis community. They permit a wider range of reaction types to be performed, such as photo- and electrochemistry, and the use of more reactive intermediates due to the possibilities of *in situ* generation and capture. One method used to quickly generate a diverse range of reactions is segmented flow, where segments of pure solvent separate individual reaction samples in a single flow reactor.^26^ This technique allowed more than 5700 Suzuki-Miyaura reactions to be performed and automatically purified over an uninterrupted 4-day process.

The subsequent work demonstrated that a similar approach could be applied to diazonium cross-coupling chemistry and parallelized across 16 reaction channels,^27^ thus increasing the output of reaction data.

Both batch and continuous flow chemistry methods can be directly coupled with a computer control system to form a closed-loop, autonomous synthesis unit.^28^ It was shown that computer control could directly utilize the Suzuki-Miyaura reaction data generated. As a result of the active learning Design of Experiment (DoE) approach, all of the products of interest were obtained in high yield without any human intervention.

### 2.2 Complexity of Chemical Reactions as a Physical Object

The challenge of predicting the reaction yield stems from the intricate interplay of numerous variables. Organic reactions, in particular, can follow diverse pathways under varying conditions, resulting in a spectrum of products with associated yields. We present the most significant influences on the experimental yield in Table 1.

**Table 1:** Factors Influencing Yield of a Chemical Reaction

**Factors Influencing Yield**	**Explanation**
Low Reactivity	Reactants may not fully react, resulting in a low yield of the desired product.
Side Reactions	Other thermodynamically possible reaction paths may be followed, leading to side products and lower yield.
Reactant/Reagent/ Catalyst Deactivation	Deactivation of reactants, reagents, or catalysts caused by other reaction system components.
Thermodynamic and Kinetic Factors	Reaction conditions (temperature, pressure, concentration, etc.) can affect the reaction rate and yield.
Contaminants	Impurities in reactants or reagents can interfere with the reaction and reduce the yield.
Sensitivity to Environment	Reactions may be sensitive to environmental factors like air, moisture, or light.
Product Degradation/ Reactivity	The desired product may be too reactive or unstable, leading to further reactions or degradation.
Product Isolation	Difficulties in isolation or purification of the product can result in a lower yield.

Determining and reporting reaction yields introduces variability, as reflected by terms such as crude yield, isolated yield, conversion yield, and selectivity. Each term conveys unique nuances of the overall yield. Specifically, the isolated yield, which factors in the purification process, often reports lower values than the crude yield due to losses during purification. Conversion yield quantifies the proportion of reactants converted to desired products, and selectivity reflects the extent to which the desired product is exclusively formed. In contrast, the crude yield provides a better estimate of the intrinsic chemical reactivity. Still, its accuracy may be compromised by the presence of contaminants, including unintended side products, in the final mixture. Thus, selecting the most relevant yield term is essential to accurately evaluate a chemical reaction accurately.

The research carried out by Murray et al.^29^ illuminated the numerous factors that significantly impact the results of chemical reactions. Their results indicated that understanding all of the variables influencing a Suzuki reaction for a single pair of reactants would require an astonishing six billion experiments. These findings highlight the deep complexity and challenges scientists face in unraveling the intricate details of chemical reactivity.

Overcoming these challenges requires a strong partnership between synthetic chemists and chemoinformaticians. Combining essential knowledge about molecular reactivity, properties of all components, and their interactions is essential for accurate predictions. The presence of reliable, high-quality data is a fundamental element driving progress in predicting yields for chemical reactions.

### 2.3 Data Storage Formats

Data curation and storage in the field of chemistry continue to be focal points of in-depth discourse, bringing together chemoinformatics specialists, chemists, and machine learning experts to discuss nuances in reaction preprocessing. Among the array of formats available for molecular data storage, three-dimensional (3D) formats such as MOL, SDF, and MDL RXN stand out for their level of detail and clarity in representing molecular structures. Yet, despite their detailed nature, they do not enjoy the same widespread acceptance as one-dimensional (1D) and two-dimensional (2D) string-based molecular representations. The need for nontrivial preprocessing further reduces their use in machine learning tasks.

The Simplified Molecular Input Line Entry System (SMILES) format,^30^ commonly employed in machine learning, holds attributes like widespread acceptance, user-friendliness, and legibility. However, its use comes with inherent challenges such as nonstandardized representations, difficulties in depicting complex metalorganic compounds, and the possibility of generating chemically inconsistent yet technically valid strings. Sodium hydroxide, for instance, can be denoted as [*Na*+].[*OH*−]. Yet, it could also be represented as [*Na*]*O*, *NaOH*, or *O*.[*NaH*], among other possible variants, some of which could be treated as invalid entries in most chemoinformatics packages, such as RDKit,^31^ for example. These discrepancies can introduce ambiguity and make data preprocessing more complicated.

**Figure 1:**
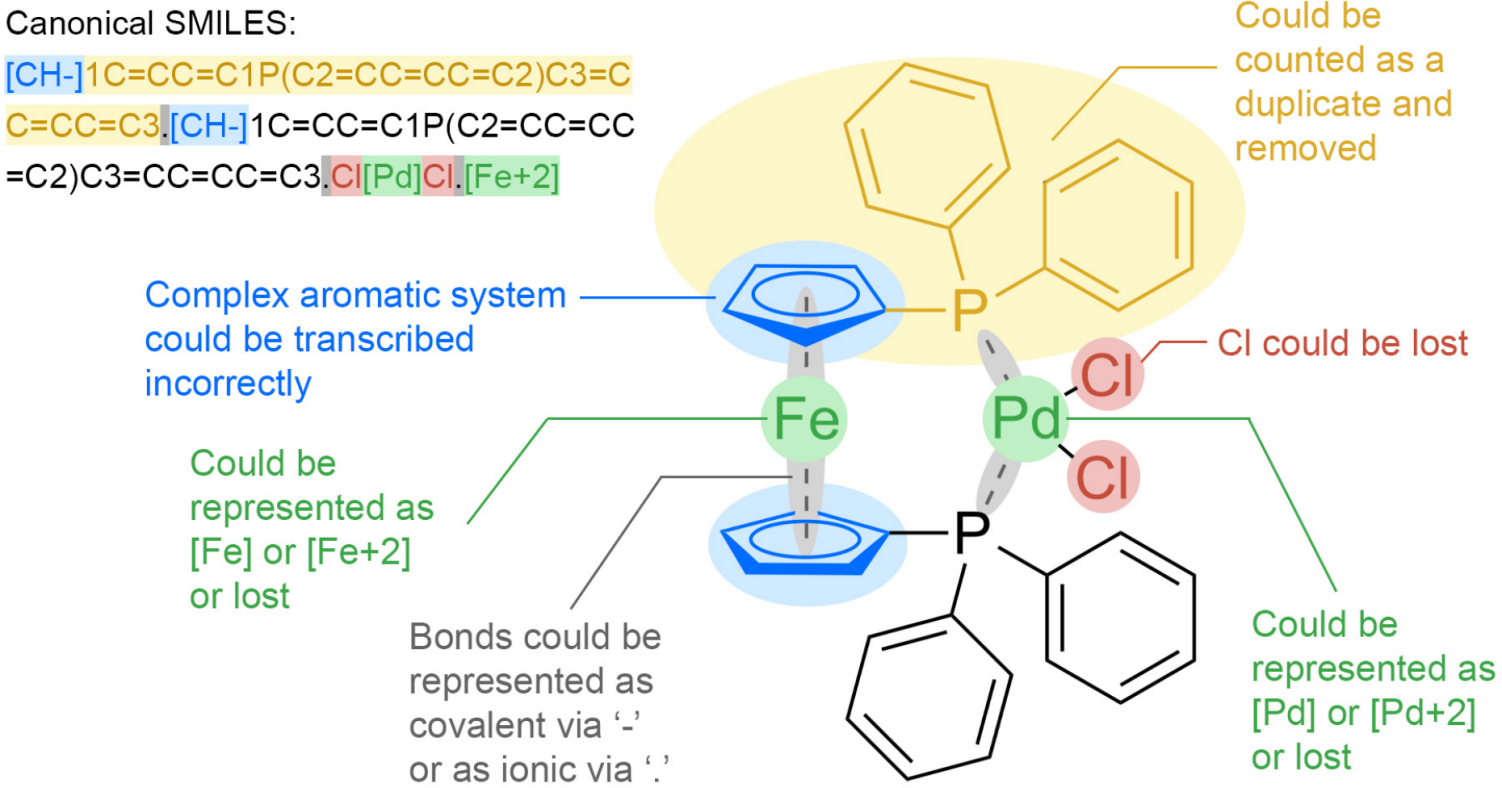
Illustration of potential inaccuracies in the depiction of molecules using PdCl_2_(dppf) as an exemplar. This Pd-containing catalyst finds extensive application in diverse couplings, encompassing Suzuki coupling and Buchwald-Hartwig reactions.

The limitations of SMILES representation become more apparent in the context of complex entities, for example, transitional metalorganic compounds,^32^ such as palladium catalysts often employed in Buchwald-Hartwig coupling reactions. Molecules such as *Pd*(*Ph*_3_*P*)_2_^2+^ and *Pd*(*Ph*_3_*P*)_4_ might be erroneously represented in a similar fashion using SMILES, introducing potential discrepancies into the data. In addition, palladium complexes can be denoted in neutral and ionic forms, raising the likelihood of generating incorrect SMILES notations, which can adversely impact the molecular encoding. Moreover, during data storage, SMILES representations of diverse palladium catalyst ligands could mistakenly be classified as duplicates, potentially resulting in unintended exclusions from the final data set. We visually illustrate their problems in Figure 1.

Efforts to address the issues of nonuniqueness and invalid SMILES representations led to the development of Self-Referencing Embedded Strings (SELFIES),^33^ designed to produce only valid molecular structures. Yet, even with advancements in the realm of SELFIES,^34^ its adoption remains limited, and it has not completely resolved the current issues associated with complex molecules. The work by Varnek’s team^35^ offers a comprehensive overview of the prevalent challenges in reaction data standardization, highlighting issues like inaccurate data recording and parsing. While their proposed data curation pipeline is thorough, it may be deemed overly broad for specific tasks such as predicting reagents or stereochemistry given its procedures for removing ions, stereochemistry, and radicals.

### 2.4 Data Sources, Reaction Data Sets

The primary task for successful modeling of chemical reaction yields is to select a data set for the purpose. Benchmark data sets frequently employed in yield prediction include the Buchwald-Hartwig coupling High-Throughput Experimentation (Buchwald-Hartwig HTE or BH HTE) data set,^36^ the Suzuki coupling HTE data set,^26^ and the United States Patent Office (USPTO) extracted data set.^20^ The first two data sets originate from high-throughput screenings that aim at finding the best reaction conditions and represent a comprehensive exploration of many combinations of reaction variables. In contrast, the USPTO data set is gathered by text-mining patents from the United States, covering publications from 1976 to September 2016, and therefore encapsulates sparse and diverse chemical reaction data.

The HTE data sets and patent data sets display distinct differences in their content and quality. While HTE data sets primarily focus on a specific segment of the chemical reaction space, they provide detailed information related to certain reaction templates tested with various selected precursors, such as reactants, solvents, bases, catalysts, and the like. On the other hand, reactions found in patents encompass a much wider scope in the chemical landscape, the extent and nuances of which will be further discussed in section 4.2.

Other currently available reaction databases include commercial products such as CAS, Reaxys, and Pistachio. Open Reaction Database (ORD), an open-access initiative,^37^ was introduced recently, aiming to curate and host reaction data in a format tailored for training machine learning models, and the different data sets in this database are list in Table 2. A significant feature of this initiative lies in its potential as a hub for sharing industry-specific data sets, which might otherwise stay confined and not be accessible to the broader scientific community. Regarding data quality, HTE data sets have the advantage of representing reactions and yield measurements carried out using the same analytical equipment, ensuring consistent and high-quality data collection.^38^ On the other hand, yields documented in patents and journal papers are measured using a range of equipment used by different institutions. Moreover, the original patent documentation frequently omits essential details, such as certain reagents or specific reaction conditions. The inherent challenges of text mining only add to these issues, often leading to noisy and incomplete data sets. Still, it needs to be acknowledged that chemists working on individual experiments most likely take more care in the purification and analysis of reactions compared to the massive workup that is required for HTE.

**Table 2:** Datasets^a^ with Available Yield Information Available for Download from ORD^37^ and Two Proprietary Datasets.

**Dataset**	**Number of reactions**
Synthesis of islatravir by biocatalytic cascade^39^	3
Copper-Catalyzed Enantioselective Hydroamination of Alkenes^40^	3
Development of an automated kinetic profiling system with online HPLC for reaction optimization^41^	7
Coupling of a-carboxyl sp3-carbons with aryl halides^42^	24
Building a Sulfonamide Library by Eco-Friendly Flow Synthesis^43^	39
Microwave-assisted Biginelli Condensation Data set^44^	48
Deoxyfluorination screen^45^	80
Chemistry informer libraries: a chemoinformatics enabled approach to evaluate and advance synthetic methods^46^	90
Imidazopyridines data set^47^	384
Linking Mechanistic Analysis of Catalytic Reactivity Cliffs to Ligand Classification^48^	450
AstraZeneca Electronic Lab Notebook (AZ ELN 750)^49^	750
Photodehalogenation HTE^50^	1152
HTE Pd-catalyzed cross-coupling screen^25^	1536
Nano CN PhotoChemistry Informers Library^51^	1728
NiCOlit^52^	1752
Predicting reaction performance in C-N cross-coupling using machine learning (Buchwald-Hartwig HTE)^36^	4312
A platform for automated nanomole-scale reaction screening and micromole-scale synthesis in flow (Suzuki HTE)^26^	5760
**Reaxys (nonpatents)**(53)	∼1.7M
USPTO curated from ORD^20^	∼1.7M
**Pistachio**(54)	6.9M

a Proprietary datasets not included in ORD are highlighted in bold.

**Figure 2:**
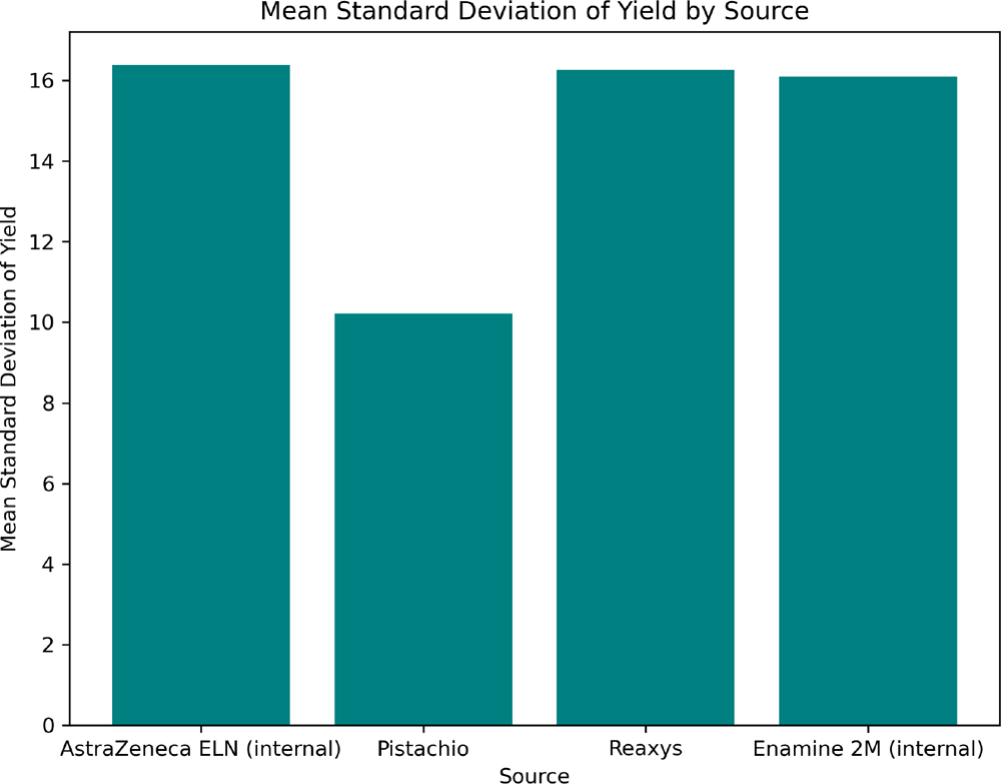
Plot illustrates that the mean yield deviation between the inner data and Reaxys data sets is consistent, but the Pistachio data set exhibits a lower standard deviation (std) in comparison.

To highlight the variability of the yield of a chemical reaction as a numeric metric, we investigated the available data from different sources. HTE data sets were not included in this analysis because there are very few to no records of the same reaction. Reactions recorded in these data sets could be executed multiple times, with each experiment recorded. We analyzed the mean and standard deviation of yield in the available data sets to assess the feasibility of regressive yield modeling and better assess its accuracy expectations. To focus on successful reactions and understand how the yield deviates in such cases, we filtered out reactions with a yield of 0. Additionally, we excluded pairs of yield values of kind [0.0, *value*] under the assumption that zero yield is likely to be associated with small-scale test reactions executed without product isolation. Also, we filtered out values different by ±1% due to potential rounding errors. The results, Figure 2, revealed a standard deviation of around 16% in more general data sets combining many reaction types. This indicates that the general reactivity model faces additional data-related challenges, and its root-mean-square error (RMSE) can not be lower than 16% in this case.

### 2.5 Data Problematics

**Figure 3:**
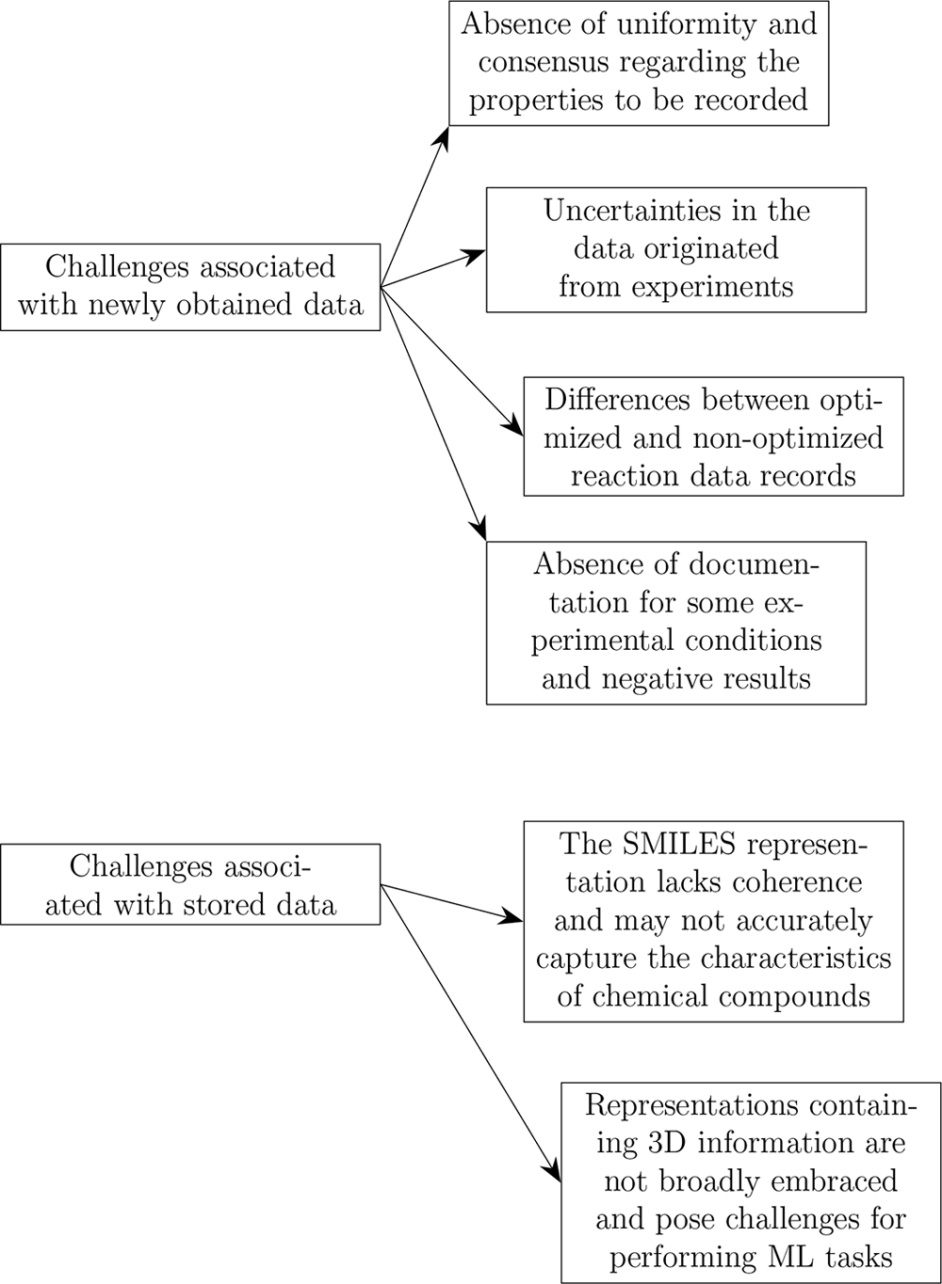
Main problems that chemoinformaticians are facing when working with chemical data sets.

We summarize the most popular problems among chemoinformaticians working with the chemical reaction data in Figure 3. In what follows, we outline some problems in more detail.

First, we need to address the fact that the availability of yield data is far from guaranteed for reported reactions. Often, only the major product is recorded, and any data on side products is missing. And if the side products are recorded, the distribution might not be normalized to 1. Thus, much reaction data cannot be used for yield models or need extensive preprocessing.

Schwaller et al.^21^ observed that the USPTO includes data from both subgram and gram reaction scales. A lower reaction scale is typically indicative of “test reactions”, preliminary experiments conducted to assess the feasibility of the reaction. Conversely, higher-scale reactions, often termed “optimized” reactions, are usually accompanied by an exhaustive exploration of the reaction condition space to pinpoint the conditions yielding the maximum product.

Fitzner et al.^55^ shed light on biases and the diversity present within chemical literature, pointing out the inherent shortcomings in the contemporary state of reaction data. Through an extensive analysis of over 62,000 Buchwald-Hartwig couplings from multiple databases, they furnished data-driven guides. These guides not only recommend reaction conditions but also aid in identifying less common ligands that demonstrate optimal performance when aligned with specific substrate properties chosen by users.

In their study, Schleinitz et al.^52^ carried out a curated extraction of Ni-catalyzed reactions, underscoring the importance of thorough data extraction from scholarly articles and optimization tables that support reaction optimization experiments. Furthermore, they benchmarked a range of cutting-edge machine learning methods, shedding light on the evident selection bias in published works and highlighting the notable lack of reported negative data.

Strieth-Kalthoff et al.^56^ in their recent study also study biases in reported reaction data. They discussed mainly three sources of bias: experimental errors, experimental selection bias, and result reporting bias. By modeling these sources of biases, they could conclude that it is predominantly the interplay between the sparsity of the data and the lack of negative data that restricts the possibility of deriving predictive models for chemical reactions.

As highlighted in the editorial by Maloney et al.,^57^ there is a pronounced deficiency in the reported negative reaction data. They point out that many High-Throughput Experiments (HTEs) conducted in academia often do not make it to machine-readable formats. Moreover, researchers presenting novel reactions in their publications frequently omit to mention the unsuccessful trials that paved the way to discovering the conditions for successful ones.

Maloney and coauthors propose a more granular differentiation of unsuccessful experiments, dividing them into three specific categories as follows.

- Experiments with neither remaining starting material nor detectable product.
- Experiments where the majority, if not all, of the starting material remains unreacted.
- Experiments not conducted as initially planned.

Having access to such detailed negative reaction data would not only allow for a clearer distinction between unreactive combinations and those that are overly reactive, leading to intricate mixtures, but also aid in identifying reactions that deviate from best practices. This would enable a more accurate association between the failed experiments and the systems’ inherent reactivity.

The significance of negative reaction data, along with other experimental details that are often omitted or inconsistently recorded in conventional publication templates, was emphasized in a recent review.^58^ Among various considerations, the authors argue that compared to other domains, such as crystallographic or NMR data, organic synthesis lacks a community-accepted standard for reporting reaction information. In an initial attempt to address this issue,^59^ the authors proposed the XDL markup language format, designed to capture comprehensive experimental details, including the timing of additions, temperature, and standard types of chemical equipment and glassware. Consequently, reaction data reported in this format would be machine-readable and writable, allowing for the postprocessing of historical reaction data and the generation of new data through fully automated synthesis. To facilitate data extraction from the literature and convert it into machine-readable format, Qian et al.^60^ and Wilary and Cole^61^ introduced tools for automated extraction of reactions and reaction conditions from diagrams and schemes. This tool holds promise for addressing the data extraction challenges previously mentioned.

## 3 Modeling

Researchers are actively investigating diverse strategies for chemical reaction yield prediction, broadly categorized into local and global approaches, and closely linked to the scale of data employed for modeling. The former encompasses traditional fingerprint-based methods tailored to precision within specific reactions, while the latter involves cutting-edge Deep Learning techniques capable of handling large databases. This section offers a comprehensive overview of these strategies, highlighting their respective strengths and challenges in predicting reaction yields.

**Figure 4:**
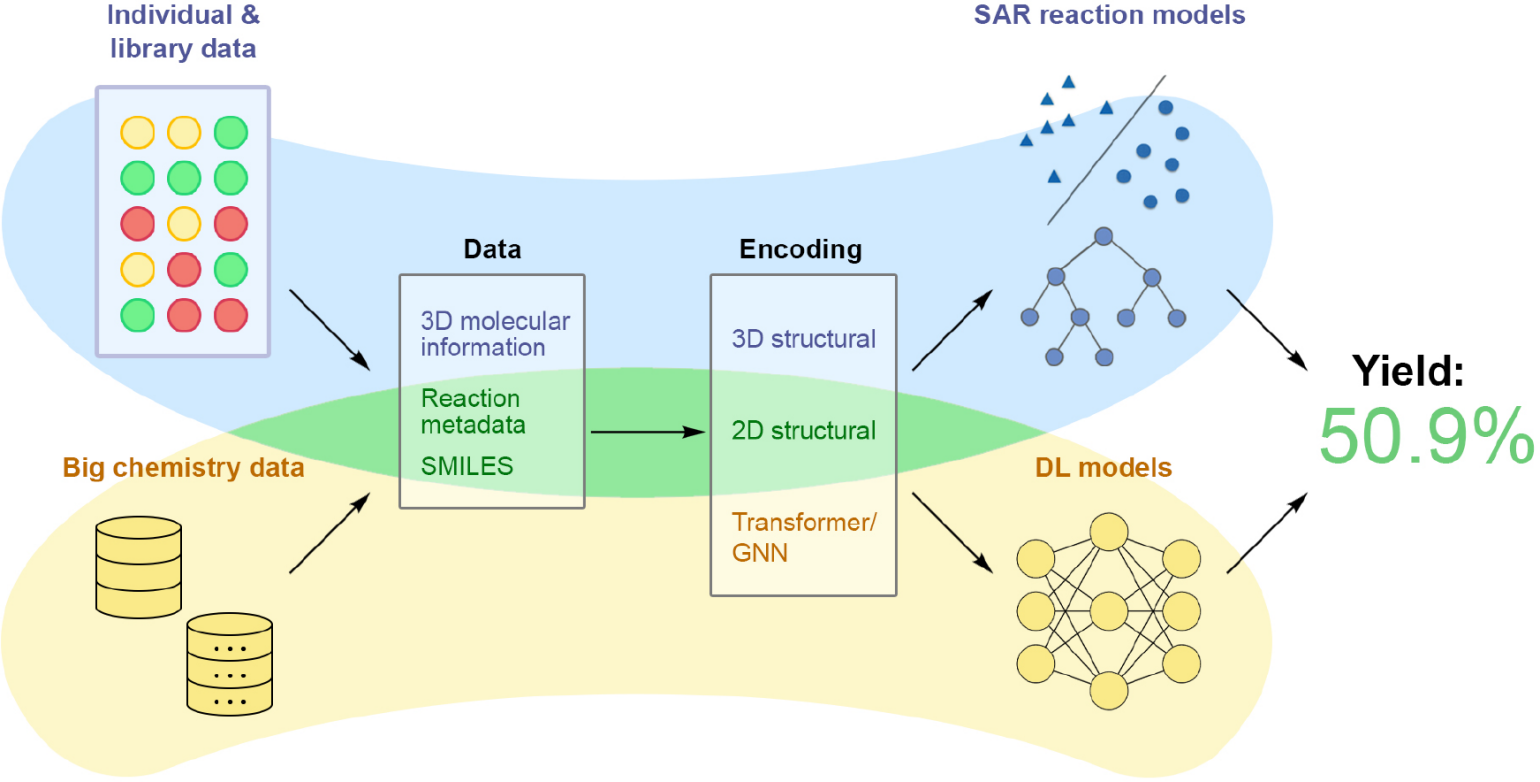
Two current State-of-the-Art approaches in yield prediction. The top row illustrates a more classical approach, while the bottom row illustrates a modern approach.

Closely associated with the scale of data used for modeling, the chemical reaction yield prediction can be categorized into two groups. The first group encompasses traditional fingerprint-based methods reminiscent of those employed in quantitative structure–activity relationship (QSAR) modeling for smaller chemical systems. The second, a more recent area of research, involves Deep Learning techniques that harness language model encodings and graph encodings, typical for big data tasks (Figure 4). We begin by discussing the well-established fingerprint-based methods, many of which have assimilated novel features. Thereafter, our attention shifted to cutting-edge Deep Learning techniques. This review is intended to deliver a comprehensive overview of the prevalent strategies in the domain, underscoring their respective merits and potential challenges.

The first approach focuses on smaller reaction spaces, tailoring models to optimize specific experiments and thus aiming for precision within a particular context. Typical benchmark data sets employed here include the Buchwald-Hartwig and Suzuki HTEs. Integral to this method is feature analysis; by identifying crucial features, scientists intend to boost both the accuracy and the interpretability of their models.

On the other hand, the second approach navigates larger data sets, deploying more complex models capable of handling vast volumes of data. The key objective here is to develop a comprehensive reactivity model that can predict yields across a diverse range of reaction types.

### 3.1 Reaction Encoding

The history of fingerprint encoding can be traced back to the 1960s with the creation of the first substructure-based fingerprints, notably the Morgan fingerprints.^62^ Over the decades, these substructure-centric fingerprints have retained their prominence, capturing the critical chemical attributes of a compound. More recently developed fingerprints harness the capabilities of pretrained Deep Learning models, including Graph Neural Networks (GNN) and Large Language Models. The most widely used examples of fingerprints are shown in Table 3.

**Table 3:** Most Common Reaction Encodings for Yield Prediction

**Reaction encoding**	**Short description**
Methods developed specifically for the reaction encoding	
RXNFP^66^	Developed to encode SMILES using a pretrained BERT model fine-tuned on Pistachio.
DRFP^67^	A binary fingerprint based on the symmetric difference of two sets containing the circular molecular n-grams generated from the molecules listed left and right from the reaction arrow in SMILES.
Graph-based encodings	Chemprop^68,69^ implemented the support of CGR^63^ and uses the pseudomolecule for message passing.
Encodings calculated for the individual components of the reactions	
DFT fingerprints	Include various features, calculated for each molecule using Quantum Calculation software.
Structural fingerprints (ECFP, Rdkit structural)	Fingerprints that are based on the structure of the molecule and calculated structural features,^70^ use SMILES.
Graph-based encodings	AttentiveFP,^71^ MoGAT.^72^

CGR, or Condensed Graph of Reaction, is a representation that combines reactants and products into a single 2D graph, encompassing both conventional and changing bonds. Developed by Varnek and colleagues,^63^ the CGR approach encodes molecular structures using fragment occurrence in a matrix. It offers a superposition of reactant and product molecules, describing alterations in atoms and bonds, reminiscent of the transition-state concept.^64^ This approach has seen increasing adoption in recent cheminformatics research, leading to the creation of an open-source toolkit by Varnek and colleagues to facilitate wider CGR utilization.^65^ However, it is worth noting that this approach relies on correct reaction atom mapping, a current challenge in the field.

Apart from fingerprints and graph representations of the reactions, the SMILES representation discussed in section 2.3 can be used directly with language models.

### 3.2 Low-Data ML & Active Learning

The optimization of chemical reactions via high-throughput experiments often demands significant resources. This has led researchers to investigate alternative strategies, especially active learning, to navigate situations with limited data. The essence of these strategies is to glean maximum insights from such narrow data sets by pinpointing and harnessing the most important and informative features. The data sets derived from a single experimental setup, usually HTE, are referred to by us as “low-data” experiments. Usually, the settings of the experiment are as such: the number of data points derived from a single experiment does not exceed ten 000 single reactions.

In a pioneering attempt at yield prediction using machine learning, Ahneman et al.^36^ tackled the problem on the Buchwald-Hartwig HTE data set by leveraging multiple density functional theory (DFT) calculated descriptors and a range of ML techniques, including Random Forest and simple Neural Networks, reaching Root Mean Squared Error (RMSE) 7.8% and *R*^2^ value of 0.92 for the best Random Forest Model (RF) for 70/30 train/test random split set. For leave-one-additive-out, the average RMSE was 11.3% and *R*^2^ 0.83. However, their methodology was later scrutinized by Chuang and Keiser,^73^ who pointed out potential redundancy and the minimal informational value of the DFT features, especially considering their computational cost since they reached RMSE of 7.9% and *R*^2^ of 0.91 with random features for the same splitting. Despite this criticism, subsequent research by Żurański et al.^74^ indicated that DFT features could indeed offer valuable insights into reaction mechanisms and exhibit enhanced generalization across diverse reaction spaces, demonstrating RMSE between 5 and 25% for leave-one-additive-out approach with RF. Building on this, Sandfort et al.^75^ found that a combination of features often outperforms simplistic one-hot encodings, reaching an *R*^2^ score of 0.93, while one-hot showed *R*^2^ of 0.89 on 70/30 random split of BH HTE data set. In another work, Dong et al.^76^ studied the importance of specific features in yield prediction using the SHAP (Shapley Additive exPlanations) library in tandem with XGBoost models, and SHAP usage gives an insight into the most important features, such as electronic descriptors of aryls and ligands. Also, the XGBoost model showed a good performance on the BH HTE data set with a 90/10 random split of RMSE 5.01% and *R*^2^ of 0.97, on the leave-one-additive-out the XGBoost model outperformed RF.

Johansson et al.^77^ demonstrated that learning just a fraction of the HTE data set can be enough to achieve high prediction accuracy. They employed various models, including simple neural networks, complex neural networks, random forests, and Bayesian matrix factorization models. The study utilized an uncertainty-based active learning strategy known as Margin and reached an area under the receiver operating characteristic (AUROC) of 0.9 using only selected 10% of the BH HTE data set. Prior work on active learning for predicting outcomes of Suzuki coupling was conducted by Eyke et al.,^78^ although Active Learning was not outperforming random learning until the Active Learning approach had less than 17% of the Suzuki data set. The authors employed this approach to optimize the number of experiments required to learn the essential features of reactions.

Kexin et al.^79^ propose MetaRF, an attention-based random forest model optimized by a meta-learning framework for few-shot yield prediction, and introduce a dimensionality reduction-based sampling method to improve few-shot learning performance. The methodology shows the performance of *R*^2^ of 0.7738 for leave-one-ligand-out and shows *R*^2^ of 0.648 using only selected 2.5% of the BH HTE data set.

Haywood et al.^80^ compared different Support Vector Regression (SVR) kernels with different descriptors, including DFT calculated and structural for the BH HTE data set, and found that structural fingerprints perform slightly better than the DFT ones, with RMSE of 17.4% and *R*^2^ of 0.51 for the structural and RMSE of 23.1% and *R*^2^ of 0.24 for DFT in leave-one-additive-out setting. The authors also attempted to assess the model applicability domain, investigating leave-one-aryl halide-out, leave-one-base-out, and others. They claim that the HTE data need to be more diverse to allow building a better generalizable model. Using different fingerprints, Bayesian modeling, and the BH HTE data set as a benchmark, Ranković et al.^81^ optimized the selection of additives that lead to higher-yielding reactions. The authors highlighted that employing Bayesian optimization modeling should facilitate the reaction optimization process using HTE. The development of a chemoinformatics workflow for achieving high yields in Buchwald-Hartwig couplings was explored in a study by Fitzner et al.^82^ The investigation focused on developing a new descriptor to reduce the number of experiments necessary for capturing critical information using an active learning approach; to assess the success of the descriptor, they used the Spearman coefficient ρ that takes values between −1 and 1, and their custom XGBoost model reached a value of 0.5. This research also studied the obstacles preventing the achievement of good results in modeling Buchwald-Hartwig C-N coupling reactions.

Reker et al.^83^ developed LabMate.ML which is a computational framework for leveraging random, unbiased experiments to navigate the selected reactivity space employing adaptive machine learning.

Collectively, the studies listed above highlight the active learning strategies employed in yield prediction, the importance of feature selection and engineering, and the efforts made to optimize experimental workflows and effectively capture information from the limited data for various types of chemical reactions.

### 3.3 Big-Data Deep Learning Models

In Deep Learning (DL), featurization for the reactions is done using either SMILES representation as strings of tokens or molecular graph representation with nodes and edges. We refer to “big-data” as the data derived from multiple experiments of the same reaction type and more general data sets that combine multiple reaction types derived from diverse sources. Usually, the number of data points exceeds tens of thousands.

Yield-BERT, developed by Schwaller et al.,^21^ was a groundbreaking model that successfully implemented the Transformer architecture^84^ and used SMILES representation as an input, reaching *R*^2^ of 0.951 for random 70/30 BH HTE, and RMSE of 12.07% and *R*^2^ of 0.81 for Suzuki data set on 70/30 random split. Data augmentation played a pivotal role in enhancing the capabilities of Yield-BERT, especially in situations with sparse data sets. This enhancement increased the model’s robustness and endowed it with the capacity to assess the uncertainty inherent in yield predictions. In a related study, Baraka et al.^85^ employed a Multimodal Transformer-based Model for predicting yields in Buchwald-Hartwig and Suzuki-Miyaura reactions, reaching *R*^2^ of 0.959 for BH HTE on 70/30 random split, RMSE of 5.5 and *R*^2^ of 0.833 for Suzuki, and RMSE of 11.5 on 70/30 random split. Their findings emphasized that amalgamating diverse modalities into the prediction process can significantly improve the results for these specific chemical reactions.

For Deep Learning models that view reactions as graph entities, the most widely used frameworks are Graph Neural Networks (GNN) and Message-Passing Neural Networks (MPNN).^86^ As an example of this, Sato et al.^87^ merged MPNN with self-attention mechanisms for yield predictions; the model resulted in *R*^2^ of 0.972 when using Mol2Vec^88^ atom embedding for BH HTE data set in 70/30 random split. Their work highlighted the importance of particular atoms within the model’s calculations. However, their method encountered challenges predicting outcomes for certain chemotypes within the benchmark data sets. In another study, Youngchun et al.^89^ employed Message-Passing Neural Networks to enable uncertainty-aware learning of reaction yields using the benchmark data sets, introducing the parameter λ which is responsible for the relative strength of two objectives (minimize the conventional mean-squared error and maximization of the log-likelihood over the training data set). With λ = 0.1 the model reached *R*^2^ score of 0.974 for a 70/30 random split for the BH HTE data set. They have shown that higher predicted variances are often concomitant with higher prediction errors, which provide a criterion to selectively dismiss certain predictions. In another work, Saebi et al.^49^ tested various techniques and reported the YieldGNN. This model performed well on High-Throughput Experimentation (HTE) data, *R*^2^ of 0.957 for YieldGNN with no chemical features. Nonetheless, its efficacy deteriorated when tested on a chemically diverse data set from AstraZeneca’s Electronic Lab Notebooks (AZ ELN), *R*^2^ of 0.049.

In the context of yield prediction, the Transformer architecture has demonstrated a potential benefit over the GNN models. This success opens avenues to explore the interpretability of these networks, in particular, to understand their internal mechanisms of “interpreting” reactions. This was exemplified by the creators of Yield-BERT, where they compared the model’s learned attention patterns with reaction mapping.^66^

Neves et al.^90^ introduced a novel technique that augmented the Transformer model standard SMILES encoding with reaction equivalents. Their investigation demonstrated the potential advantages of using this approach to improve industrial synthesis operations. Their methodology employed a binary classification, where reactions yielding 5% or less were labeled as unsuccessful. Uncertainty estimates were analyzed for the successful and unsuccessful classes. When the model was validated on the internal ReactLake reaction database using a temporal split, it was shown that 52.8% of negative reactions can be correctly flagged and thus experimentally avoided. The overall model’s performance was satisfactory, with a recorded receiver operating characteristic (ROC) area under the curve (AUC) value of 0.76 in experimental validation.

Yarish et al.^91^ developed the directed message-passing neural network (RD-MPNN) yield prediction models, which they tested on Enamine’s proprietary reaction data. Their binary classification model showed a commendable ROC AUC of 0.78. When extended to a ternary classification setting, the model displayed an accuracy of 0.51 across multiple reaction classes. Interestingly, the RD-MPNN’s performance was on par with the leading results obtained on the BH HTE benchmark data set and surpassed other models when tested on the Suzuki data set, with a coefficient of determination (*R*^2^ 0.93 for BH HTE, RMSE 10.35%, *R*^2^ 0.86 for the latter). Also, the authors performed an analysis of erroneous predictions. They identified key challenges, including issues associated with product isolation by chromatography and reduced yields due to steric hindrance and competing side reactions.

Jian et al.^22^ developed a unique SMILES-based model for yield prediction. Based on a bespoke tokenization procedure, a long short term memory (LSTM)-based architecture, and data from both USPTO and proprietary sources, they could obtain an RSME of around 20%.

## 4 Benchmarking

In this section, we undertake a series of experiments aimed at illustrating typical examples of yield or reactivity modeling that encompass both medium- and large-scale data modeling scenarios. Our experiments delve into the underlying complexities of the Buchwald-Hartwig reaction, which significantly impact the modeling process and the feasibility of modeling in general. This section is structured into two cases: “successful” and “unsuccessful”, corresponding to modeling using the HTE Buchwald-Hartwig data set and modeling with USPTO and Reaxys Buchwald-Hartwig reaction selections, respectively. Although we limit our experiments to Buchwald-Hartwig reactions in this report, we believe that the learnings can be transferred to other reaction classes that are similarly complex. For reaction classes with less complexity, the modeling might be more successful. We chose to work with Buchwald-Hartwig reactions, because it is a very common reaction in the pharmaceutical industry that consequently has received attention in the modeling community.

### 4.1 A Successful Case Example: HTE Buchwald-Hartwig Amination Yield Prediction

**Figure 5:**
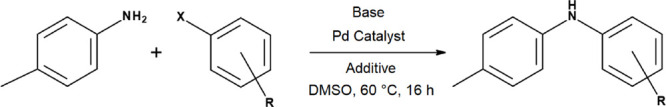
Buchwald-Hartwig Amination Reaction^36^

Ahneman et al. made a significant contribution to the yield prediction field with their groundbreaking work on the Buchwald-Hartwig reaction, Figure 5, within a high-throughput experimentation framework.^36^ The reaction data set in this work was generated using high-throughput experimentation in three 1536-well plates, enabling exhaustive variation of reaction components. The initial data set retained 3955 reaction data points after eliminating essential control experiments and reactions involving the additive 7. This work used 15 aryl halides, 23 additives, four palladium catalysts, and three bases overall.

Ahneman et al. used a range of molecular properties derived from DFT-level theory simulations of the reaction components as descriptors. These descriptors included the highest occupied molecular orbital (HOMO) and lowest unoccupied molecular orbital (LUMO) energies, NMR shifts, dipole moments, electronegativities, and others. The authors evaluated several machine learning models, ranging from linear models, k-nearest Neighbors (k-NN), Random Forest Regression, Support Vector Regression, and Bayes generalized linear models, to a shallow Artificial Neural Network (ANN). Their findings pointed toward the Random Forest model as the top performer.

Their research, however, did not proceed without contention. Chuang and Keiser critiqued their methodology, presenting evidence that substituting the DFT descriptors with random values or adopting simple one-hot encoding yielded comparable model performances.^73^ They posited that the significance that Ahneman et al. attributed to the DFT features might have been overstated. Instead of dismissing these claims, Ahneman and co-workers acknowledged this critique. They concurred on the importance of integrating random controls in subsequent research, emphasizing its critical role in enhancing the robustness and validity of future work.^92^

**Figure 6:**
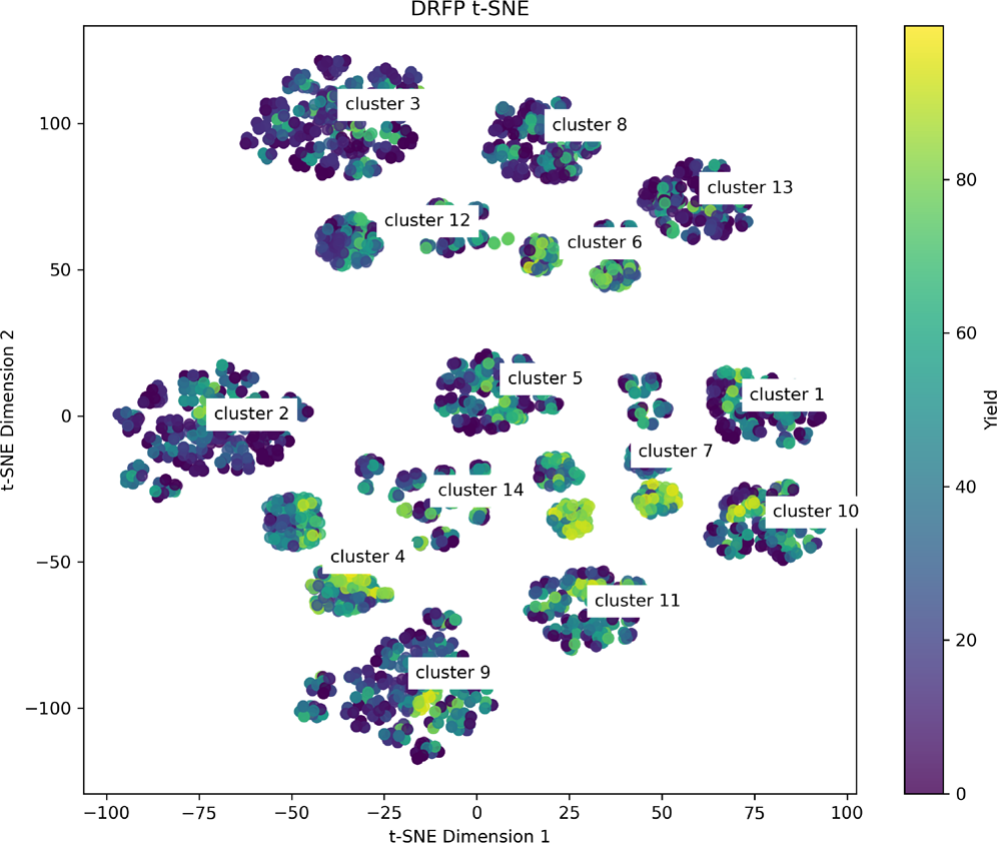
t-SNE plot for BH HTE data set, based on DRFP features. Clusterized with K-Means, number of clusters = 14.

This data set possesses several unique characteristics worth noting in the context of yield prediction. First, it contains vast, dense reaction data encompassing diverse combinations of reactants, ligands, and reagents, all annotated with the respective yield. This enables the visual representation of the data, as shown in Figure 6, clustered into different regions colored by yield. It is possible to identify areas with low and high yields from that.

Furthermore, the high data density, coupled with the subsequent cluster analysis, offered valuable insights into the scenarios where the use of specific ligands in the HTE setup resulted in suboptimal yields. A more comprehensive examination of this phenomenon was undertaken in the study by Fitzner et al.^55^

The consistent experimental setup maintained throughout the entire HTE campaign ensured the data set was conducive to accurate predictions of numerical yield values. In such a low-noise environment, the model is more capable of discerning patterns from the relevant reactions, capturing critical information from adjacent data points, and making accurate extrapolations, resulting in highly precise predictions.

Nevertheless, the constraints of the HTE data sets must be recognized. The data are bound by the specific experimental design employed, implying that the model’s predictive capability is limited to the scope of this design. Predicting the reaction outcomes for ligands or conditions absent from the data set could be unreliable or even unfeasible, given the absence of respective training data. This underlines the importance of assessing the applicability of the model domain before its deployment.

To obtain a more comprehensive understanding of the state-of-the-art approaches applied to this data set, we undertook a set of experiments to replicate existing results and evaluate the model’s generalization capabilities.

We decided to employ two modeling approaches that reflect current trends in reaction yield modeling.

- A classical tree- and kernel-based ML model utilizing reaction fingerprints.
- The Yield-BERT model, utilizing SMILES encoding, as reported in ref (21).

Reaction fingerprints (ECFP4,6,^70^ RXNFP,^66^ DRFP^67^), described previously in more detail in Table 3, were used for SVR,^93^ RFR,^94^ and Gradient Boosting Regression^95^ (GBR) models. For the modeling process, we used the Scikit-Learn^96^ Python library.

The selected model types also exemplify various Machine Learning approaches. Random Forest Regression and Gradient Boost Regression are ensemble methods; the former ensembles decide trees, while the latter ensembles weak models. On the other hand, Support Vector Regression utilizes support vector machines to learn the best-fit hyperplane to categorize the data.

We chose these different fingerprint methods to compare various approaches for encoding reactions as objects. RXNFP represents a pure data-driven encoding approach, while ECFP and DRFP represent structural approaches. This comparison allows us to gain insights into the strengths and limitations of each method in the context of yield prediction.

For embedding purposes and to avoid any possible bias connected to how different methods align the reaction components, we use the following order to build the reaction object.
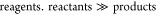


**Figure 7:**
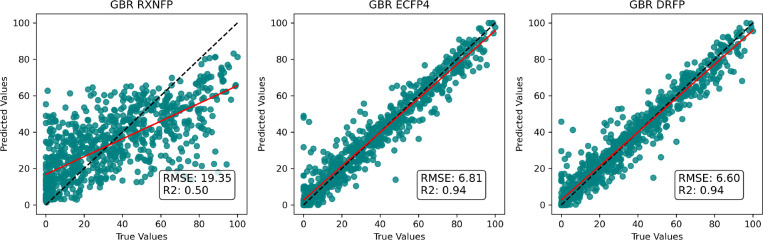
Comparison of the GBR model’s performance using different encodings and fingerprints, trained with a random 80:20 ratio and 5-fold Cross-Validation. RMSE = root-mean-square error, *R*^2^ = determination coefficient. The red line represents numpy linear fit. RFR and SVR models were excluded from the main figure for clarity, and their detailed results can be found in Supporting Information.

Initially, the models showed modest performance on a random split, as we can see in Figure 7. The results reveal that, among the simple models, the DRFP^67^ encoding exhibits the best performance, slightly outperforming ECFP4 fingerprints.

That prompted us to conduct further evaluations on the different parts of the chemical space occupied by the data set. We could see in Figure 6 the t-distributed stochastic neighbor embedding (t-SNE) dimensionality reduction performed on DRFP features and the fact that the data set nicely separates into different clusters. We decided to employ a leave-one-cluster-out validation setup with clusters defined based on the DRFP features. As summarized in Table 4, the results indicate generally satisfactory performance, albeit with some variability in clusters that may be regarded as combinations of smaller subclusters.

**Table 4:** Leave-One-Out Cluster Performance of the Gradient Boosting Regression Model Based on DRFP Features^a^

**Cluster No.**	1	2	3	4	5	6	7	8	9	10	11	12	13	14
**RMSE**	7.71	8.50	12.97	13.54	4.77	23.66	13.33	7.66	9.15	5.59	4.46	17.90	9.56	7.78
***R*^2^**	0.90	0.86	0.66	0.73	0.96	0.36	0.76	0.88	0.87	0.96	0.98	0.40	0.84	0.92
**Mean yield**	28.10	25.19	23.33	53.01	30.31	45.94	58.16	23.04	31.28	38.45	40.38	31.75	21.82	35.77

a For the visual representation of the model’s performance, see Supporting Information Figure S8.

Upon analysis of the results, it became evident that the model’s efficacy tends to diminish less when the mean of a given cluster is closer to the mean of the overall distribution. Conversely, there is a marked decline in the performance when the yield of a cluster deviates substantially from the overall mean. This indicates that the model probably struggles in predicting yields at more extreme values.

Furthermore, we investigated the model’s ability to extrapolate across reactants by executing a leave-one-reactant-out validation; specifically, focusing on aryl halides in Table 5, we could see the results of the model trained on leave-one-reactant-out. The visual results are depicted in S9. The first column row corresponds to chlorine-associated aryl halides, the middle column corresponds to bromine-associated aryl halides, and the last column corresponds to iodide-associated aryl halides. The model performs moderately well when the left-out species is a chemically reactive aryl halide. Still, the performance deteriorates when the left-out species is less reactive, for example, chlorine-containing aryl halides. This observation highlights the model’s susceptibility to variations in the chemical properties of the reactants and its potential limitation to generalize across the chemical space, even for a well-defined single chemical reaction type.

**Table 5:**
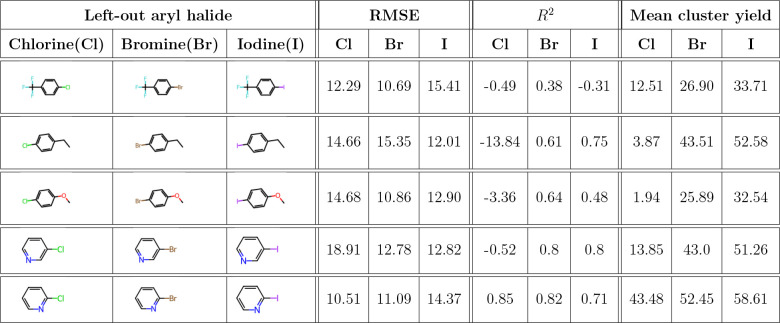
Performance of the Gradient Boost Regression Model on DRFP Features with Leave-One-Aryl Halide Out^a^

a For a graphical representation of the performance, see Supporting Information Figure S9.

We also accessed Yield-BERT properties related to the BH HTE data set, and they showed the same good results, as reported in ref (21), although on leave-one-reactant-out it showed better performance than simple models. For more information, see S7.

### 4.2 An Unsuccessful Case Example: Diverse Data Sets Buchwald-Hartwig Amination Yield Prediction

In this section, we present a case example that illustrates the challenges of yield prediction and emphasizes the importance of advancing our knowledge in condition encoding as well as enhancing the prediction methods overall. The following example showcases various aspects of yield prediction, underscoring the complexity involved. Furthermore, it is important to acknowledge that this task pertains to a broader reactivity modeling endeavor. As in the previous section, we continue focusing on Buchwald-Hartwig amination as one of the essential reactions in the pharmaceutical industry.

To obtain the reaction data, we used the web interface of Reaxys^53^ (7000 entries) and other available open-source data sets, such as AZ ELN 750^49^ (500 entries), Doyle’s HTE Buchwald-Hartwig^36^ (4000 entries), and data extracted from USPTO^20^ (6000 entries). The reactions were cleaned from duplicates and invalid entries (nonparsed via RDKit), then mapped with RXNmapper,^66^ and were classified with NameRXN.^54^ Reaction data labeled with the Next Move classes 1.3.1, 1.3.2, 1.3.3, and 1.3.4 (Chloro-, Bromo-, Iodo-, Trifluoxy-Buchwald-Hartwig Amination, respectively) was selected.

**Figure 8:**
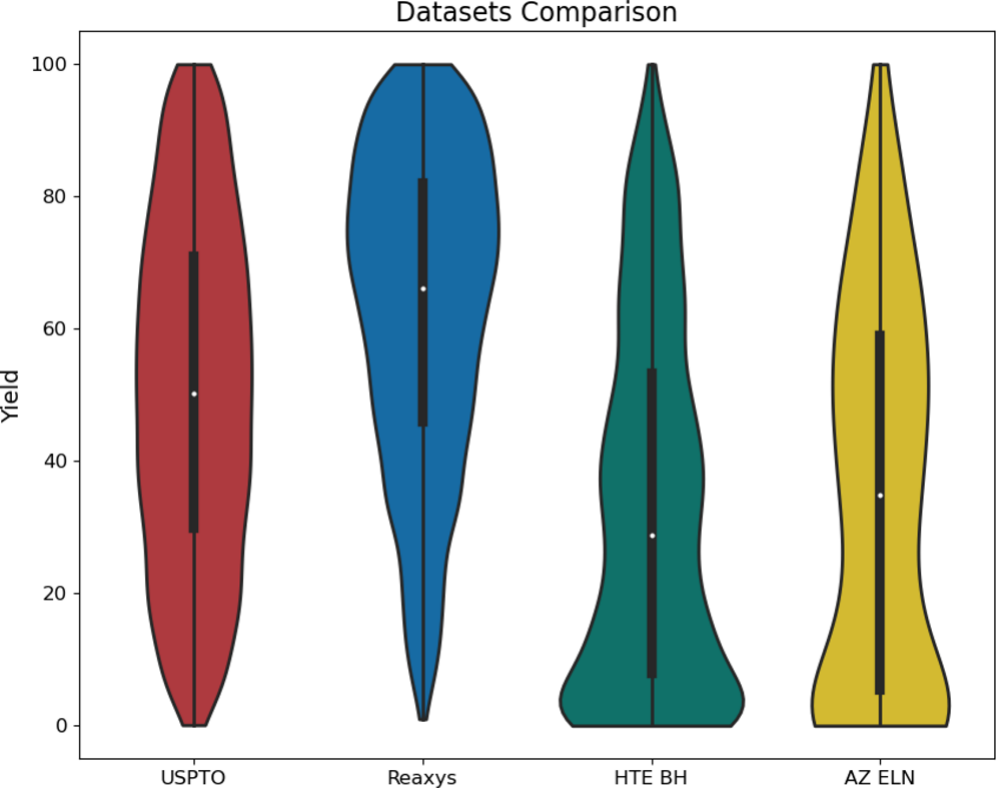
Violin plot for yield distribution for the data sets derived from public data and Reaxys.

As illustrated in Figure 8, the data sets obtained from academic experiments and industrial patents are characterized by the higher reported yields, whereas data sets derived from Electronic Laboratory Notebook records and High-Throughput Experimentation tend to often contain lower-yielding reaction data points. It is worth noting that while the U.S. Patent and Trademark Office (USPTO) data set demonstrates a similar, relatively uniform, yield distribution for this specific reaction, it is widely acknowledged that the general distribution of the USPTO data is significantly skewed toward high-yielding reactions.^21^

**Figure 9:**
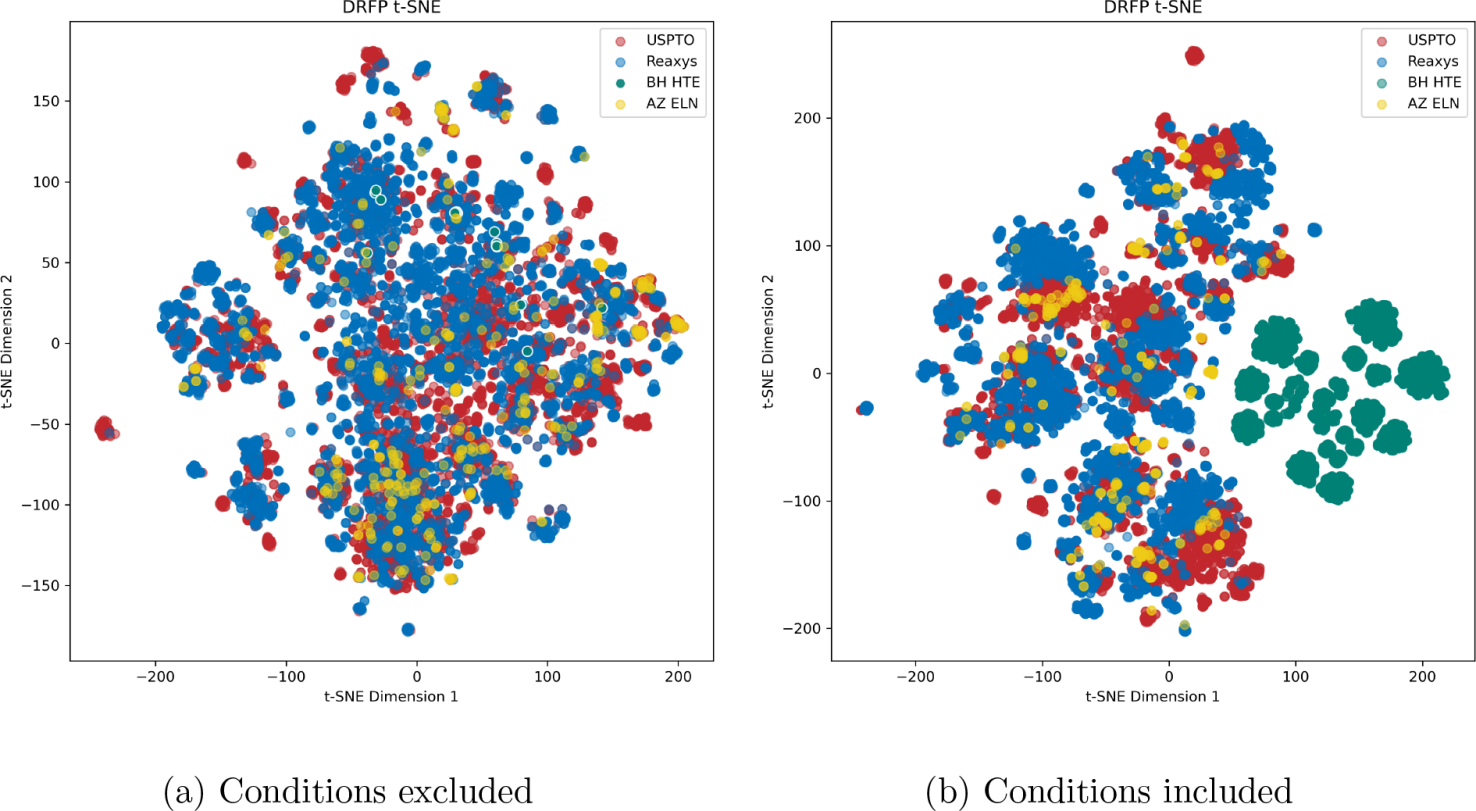
t-SNE plot depicts the distribution of reaction encodings based on DRFP representations. In (a), where all conditions are excluded, the encodings show an even distribution in hyperspace. In (b), when conditions are included, a notable separation occurs between the BH HTE data set and others. This indicates that condition representations introduce diversity, adding a layer of complexity to the encodings. We also provide a Principal Components Analysis (PCA) plot in the S4. We investigate the data recordings more in detail in S11.

Furthermore, we analyzed the distribution of reaction embeddings using t-SNE. This will serve as a qualitative analysis of the applicability domain of our models. Notably, when reagents were included, the High-Throughput Experimentation data set exhibited distinct separation in the DRFP embeddings, as illustrated in Figure 9. Conversely, Reaxys, USPTO, and AZ ELN data sets occupied dissimilar regions within the chemical space. This discrepancy could be attributed to variations in the fundamental recording of reaction components, particularly in the context of Palladium catalysts, as discussed earlier; we continue this discussion in 11. This observation leads us to propose the hypothesis that Buchwald-Hartwig reaction experiments documented in patents and articles may demonstrate a higher degree of reagent diversity compared to that of HTE experiments.

**Figure 10:**
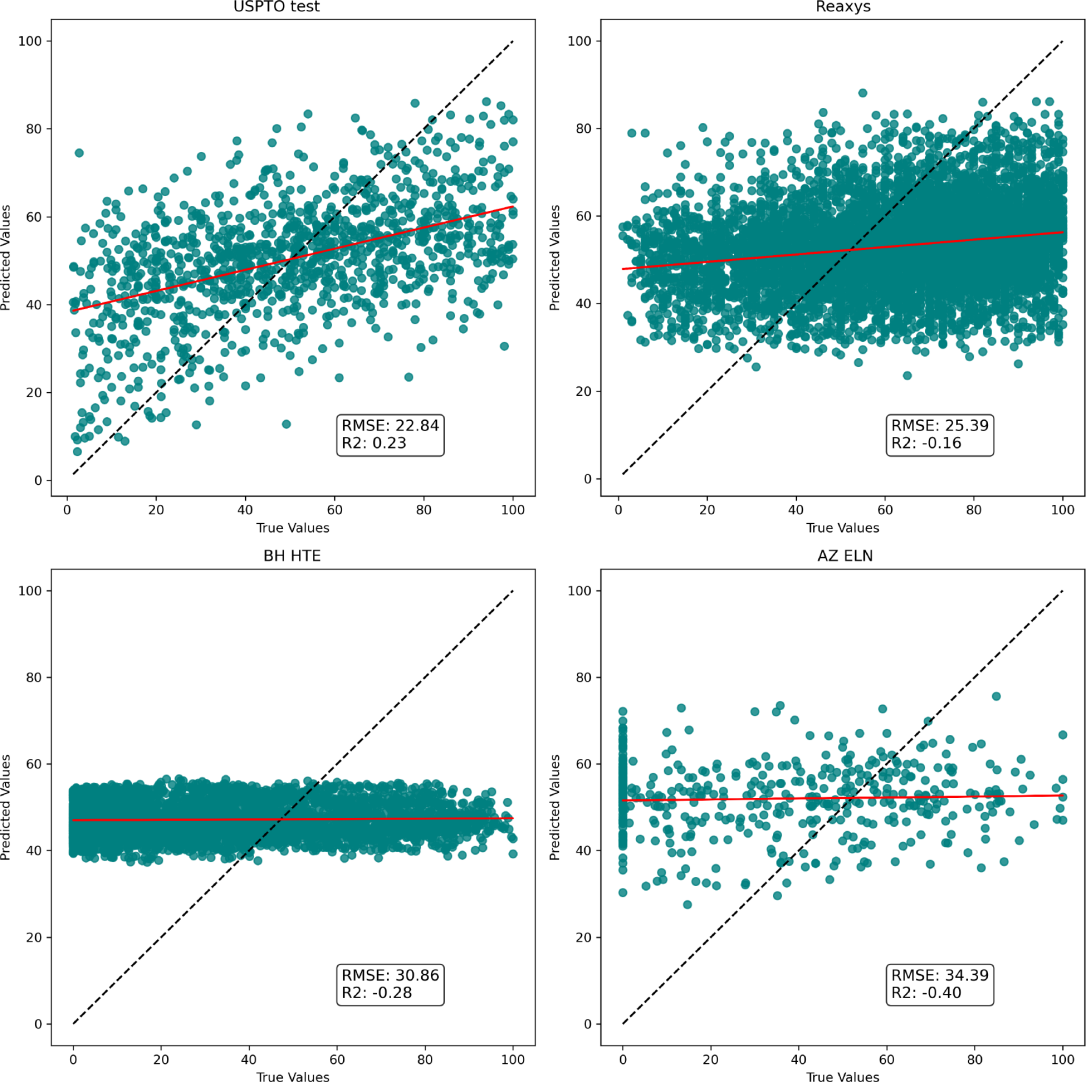
RFR model trained on USPTO Buchwald-Hartwig selection and tested on other data sets. For clarity, we show only the DRFP fingerprint performance on these plots. Other fingerprints’ performance can be found in the Supporting Information.

Using the extracted data, we modeled the model using the same procedure detailed in the previous chapter. The analysis of the model performances, as reflected in the Root Mean Square Error and coefficient of determination in Figure 10, reveals that the results achieved are unsatisfactory. When tested on real-world Buchwald-Hartwig reaction data, simple models exhibit the same performance as the more complex Yield-BERT model (see S10). This lack of performance and generalization ability could stem from various factors, including noise within the data. However, as indicated by the t-SNE plots in Figure 9, there is considerable overlap between the USPTO and Reaxys data set, indicating that the Reaxys reactions are within the applicability domain of the USPTO-derived model. The same can be said for at least the AZ ELN data but less for the HTE data set. This observation implies that current featurization methods might struggle to capture the intricate nuances inherent to specific reactions.

Consequently, the challenges in capturing the intricate chemistry inherent in this specific reaction are not unexpected. We previously delved into the issues associated with large-scale data in a dedicated section, and the results of these experiments corroborate the challenges posed by the vast and diverse chemical space.

## 5 Conclusion and Future Outlook

This review highlights that despite the progress in yield prediction methodologies, there remain significant limitations in their ability to handle diverse data sets, especially those containing chemically diverse reactant species. These challenges arise from both the data and the modeling aspects.

Data utilized for yield prediction frequently contain inherent noise and may sometimes lack crucial details necessary for precise predictions. To address this, there is a need for a standardized recording procedure that can be universally applied across academic and industrial institutions. By converting reaction conditions and procedures into a machine-readable, noise-free format, this standardization would greatly enhance the modeling process for various reaction properties that demand in-depth information.

A pivotal issue lies in the limited generalization of the model capacity. The complexity of the underlying chemical and physical mechanisms governing reaction yields is profound and perhaps more intricate than initially assumed. We believe that the challenge is not just computational but deeply rooted in understanding the fundamental principles of chemistry. In essence, the task of predicting chemical reaction yields presents a multifaceted challenge that is not solely computational. Deeper integration of the foundational principles of chemistry is crucial to advance and refine existing prediction models.

Analysis of the variance in reported yields in Figure 2 suggests that employing classification models with multiple bins can better address the complexity of the yield prediction problem, taking into account the noisy data.

The future trajectory of yield prediction development is expected to proceed along multiple paths. Due to the advancements in synthesis automation, we foresee the emergence of enhanced data sets that will incorporate a wider range of high-quality data. Concurrently, a shift toward uncertainty-based predictions seems plausible. As previously noted, the precise numerical yield can often be not feasible due to the significant noise in data. Consequently, the predicted yield has a tendency to function more as a classification label for many experimentalists. As such, broader categorizations such as excellent, good, or moderate yield might often suffice.

An intriguing avenue to explore involves detailed studies of widely used reaction classes, aiming to develop, albeit potentially more computationally intensive, chemically relevant reaction-specific descriptors. These descriptors can effectively encode reactions of the same class, enabling predictions within a specific category. This strategy demands an exhaustive analysis of the reaction mechanisms, both thermodynamic and kinetic aspects, and the unique intermediates inherent to each class. A deeper understanding of the mechanisms underlying specific reactions can be achieved, leading to the creation of an encoder that captures these unique attributes.

Venturing into these prospective areas, the domain of yield prediction is likely to benefit from higher-quality data sets, refined probabilistic predictions, and focused investigations into reaction-specific descriptors. These advancements promise to improve the accuracy and reliability of yield predictions for chemical reactions.

## Data Availability

All the source code and data sets (ReactionID for Reaxys) used to produce the reported results can be found at https://github.com/v-in-cube/YieldnotYield.

## Supporting Information Available

Additional t-SNE and PCA analysis and detailed performance results of the models trained (PDF)

## References

[ref1] Zhong Z.; Song J.; Feng Z.; Liu T.; Jia L.; Yao S.; Hou T.; Song M. Recent advances in artificial intelligence for retrosynthesis. arXiv 2023. 10.48550/arXiv.2301.05864

[ref2] Schwaller P.; Vaucher A. C.; Laplaza R.; Bunne C.; Krause A.; Corminboeuf C.; Laino T. Machine intelligence for chemical reaction space. Wiley Interdiscip. Rev. Comput. Mol. Sci. 2022, 12, 10.1002/wcms.1604.

[ref3] Tetko I. V.; Karpov P.; Van Deursen R.; Godin G. State-of-the-art augmented NLP transformer models for direct and single-step retrosynthesis. Nat. Commun. 2020, 11, 10.1038/s41467-020-19266-y. PMC7643129 33149154

[ref4] Tu Z.; Stuyver T.; Coley C. W. Predictive chemistry: machine learning for reaction deployment, reaction development, and reaction discovery. Chem. Sci. 2023, 14, 226–244. 10.1039/D2SC05089G. 36743887 PMC9811563

[ref5] Jin W.; Coley C. W.; Barzilay R.; Jaakkola T. Predicting Organic Reaction Outcomes with Weisfeiler-Lehman Network. arXiv 2017, 10.48550/ARXIV.1709.04555.

[ref6] Tomberg A.; Johansson M. J.; Norrby P.-O. A Predictive Tool for Electrophilic Aromatic Substitutions Using Machine Learning. J. Org. Chem. 2019, 84, 4695–4703. 10.1021/acs.joc.8b02270. 30336024

[ref7] Pesciullesi G.; Schwaller P.; Laino T.; Reymond J. L. Transfer learning enables the molecular transformer to predict regio- and stereoselective reactions on carbohydrates. Nat. Commun. 2020 11:1 2020, 11, 1–8. 10.1038/s41467-020-18671-7. PMC7519051 32978395

[ref8] Gao H.; Struble T. J.; Coley C. W.; Wang Y.; Green W. H.; Jensen K. F. Using Machine Learning To Predict Suitable Conditions for Organic Reactions. ACS Cent. Sci. 2018, 4, 1465–1476. 10.1021/acscentsci.8b00357. 30555898 PMC6276053

[ref9] Genheden S.; Mårdh A.; Lahti G.; Engkvist O.; Olsson S.; Kogej T. Prediction of the Chemical Context for Buchwald-Hartwig Coupling Reactions. Mol. Inform. 2022, 41, 2100294 10.1002/minf.202100294. 35122702 PMC9540548

[ref10] Beker W.; Roszak R.; Wołos A.; Angello N. H.; Rathore V.; Burke M. D.; Grzybowski B. A. Machine Learning May Sometimes Simply Capture Literature Popularity Trends: A Case Study of Heterocyclic Suzuki–Miyaura Coupling. J. Am. Chem. Soc. 2022, 144, 4819–4827. 10.1021/jacs.1c12005. 35258973 PMC8949728

[ref11] Skoraczyński G.; Dittwald P.; Miasojedow B.; Szymkuć S.; Gajewska E. P.; Grzybowski B. A.; Gambin A. Predicting the outcomes of organic reactions via machine learning: are current descriptors sufficient? Sci. Rep. 2017, 7, 10.1038/s41598-017-02303-0. PMC5472585 28620199

[ref12] Hammett L. P. Some Relations between Reaction Rates and Equilibrium Constants. Chem. Rev. 1935, 17, 125–136. 10.1021/cr60056a010.

[ref13] Aoyama T.; Ichikawa H. Neural networks as nonlinear structure-activity relationship analyzers. Useful functions of the partial derivative method in multilayer neural networks. J. Chem. Inf. Model. 1992, 32, 492–500. 10.1021/ci00009a015.

[ref14] Zheng W.; Tropsha A. Novel Variable Selection Quantitative Structure-Property Relationship Approach Based on the ik/i-Nearest-Neighbor Principle. J. Chem. Inf. Model. 2000, 40, 185–194. 10.1021/ci980033m. 10661566

[ref15] Liu H. X.; Zhang R. S.; Yao X. J.; Liu M. C.; Hu Z. D.; Fan B. T. QSAR Study of Ethyl 2-[(3-Methyl-2, 5-dioxo(3-pyrrolinyl))amino]-4-(trifluoromethyl) pyrimidine-5-carboxylate: An Inhibitor of AP-1 and NF-*κ*B Mediated Gene Expression Based on Support Vector Machines. J. Chem. Inf. Model. 2003, 43, 1288–1296. 10.1021/ci0340355. 12870922

[ref16] Svetnik V.; Liaw A.; Tong C.; Culberson J. C.; Sheridan R. P.; Feuston B. P. Random Forest: A Classification and Regression Tool for Compound Classification and QSAR Modeling. J. Chem. Inf. Model. 2003, 43, 1947–1958. 10.1021/ci034160g. 14632445

[ref17] Williams W. L.; Zeng L.; Gensch T.; Sigman M. S.; Doyle A. G.; Anslyn E. V. The Evolution of Data-Driven Modeling in Organic Chemistry. ACS Cent. Sci. 2021, 7, 1622–1637. 10.1021/acscentsci.1c00535. 34729406 PMC8554870

[ref18] Emami F. S.; Vahid A.; Wylie E. K.; Szymkuć S.; Dittwald P.; Molga K.; Grzybowski B. A. A Priori Estimation of Organic Reaction Yields. Angew. Chem., Int. Ed. 2015, 54, 10797–10801. 10.1002/anie.201503890. 26215084

[ref19] Raccuglia P.; Elbert K. C.; Adler P. D. F.; Falk C.; Wenny M. B.; Mollo A.; Zeller M.; Friedler S. A.; Schrier J.; Norquist A. J. Machine-learning-assisted materials discovery using failed experiments. Nature 2016, 533, 73–76. 10.1038/nature17439. 27147027

[ref20] Lowe D. Chemical reactions from US patents (1976-Sep2016). Artwork Size: 1494665893 Bytes Pages: 1494665893 Bytes Type: dataset, figshare. Dataset 2017, 10.6084/M9.FIGSHARE.5104873.V1.

[ref21] Schwaller P.; Vaucher A. C.; Laino T.; Reymond J.-L. Prediction of chemical reaction yields using deep learning. Mach. learn.: sci. technol. 2021, 2, 015016 10.1088/2632-2153/abc81d.

[ref22] Jiang S.; Zhang Z.; Zhao H.; Li J.; Yang Y.; Lu B.-L.; Xia N. When SMILES Smiles, Practicality Judgment and Yield Prediction of Chemical Reaction via Deep Chemical Language Processing. IEEE Access 2021, 9, 85071–85083. 10.1109/ACCESS.2021.3083838.

[ref23] Ley S. V.; Fitzpatrick D. E.; Ingham R. J.; Myers R. M. Organic Synthesis: March of the Machines. Angew. Chem., Int. Ed. 2015, 54, 3449–3464. 10.1002/anie.201410744. 25586940

[ref24] Li J.; Ballmer S. G.; Gillis E. P.; Fujii S.; Schmidt M. J.; Palazzolo A. M. E.; Lehmann J. W.; Morehouse G. F.; Burke M. D. Synthesis of many different types of organic small molecules using one automated process. Science 2015, 347, 1221–1226. 10.1126/science.aaa5414. 25766227 PMC4687482

[ref25] Buitrago Santanilla A.; Regalado E. L.; Pereira T.; Shevlin M.; Bateman K.; Campeau L.-C.; Schneeweis J.; Berritt S.; Shi Z.-C.; Nantermet P.; Liu Y.; Helmy R.; Welch C. J.; Vachal P.; Davies I. W.; Cernak T.; Dreher S. D. Nanomole-scale high-throughput chemistry for the synthesis of complex molecules. Science 2015, 347, 49–53. 10.1126/science.1259203. 25554781

[ref26] Perera D.; Tucker J. W.; Brahmbhatt S.; Helal C. J.; Chong A.; Farrell W.; Richardson P.; Sach N. W. A platform for automated nanomole-scale reaction screening and micromole-scale synthesis in flow. Science 2018, 359, 429–434. 10.1126/science.aap9112. 29371464

[ref27] Ahn G.-N.; Sharma B. M.; Lahore S.; Yim S.-J.; Vidyacharan S.; Kim D.-P. Flow parallel synthesizer for multiplex synthesis of aryl diazonium libraries via efficient parameter screening. Commun. Chem. 2021, 4, 10.1038/s42004-021-00490-6. PMC9814388 36697557

[ref28] Christensen M.; Yunker L. P. E.; Adedeji F.; Häse F.; Roch L. M.; Gensch T.; dos Passos Gomes G.; Zepel T.; Sigman M. S.; Aspuru-Guzik A.; Hein J. E. Data-science driven autonomous process optimization. Commun. Chem. 2021, 4, 10.1038/s42004-021-00550-x. PMC9814253 36697524

[ref29] Murray P. M.; Tyler S. N. G.; Moseley J. D. Beyond the Numbers: Charting Chemical Reaction Space. Org. Process Res. Dev. 2013, 17, 40–46. 10.1021/op300275p.

[ref30] Weininger D. SMILES, a chemical language and information system. 1. Introduction to methodology and encoding rules. J. Chem. Inf. Model. 1988, 28, 31–36. 10.1021/ci00057a005.

[ref31] RDKit. https://www.rdkit.org, accessed on 2022-13-07.

[ref32] Quirós M.; Gražulis S.; Girdzijauskaitė S.; Merkys A.; Vaitkus A. Using SMILES strings for the description of chemical connectivity in the Crystallography Open Database. J. Cheminf. 2018, 10, 10.1186/s13321-018-0279-6. PMC5959826 29777317

[ref33] Krenn M.; Häse F.; Nigam A.; Friederich P.; Aspuru-Guzik A. Self-referencing embedded strings (SELFIES): A 100% robust molecular string representation. Mach. learn.: sci. technol. 2020, 1, 045024 10.1088/2632-2153/aba947.

[ref34] Krenn M.; Ai Q.; Barthel S.; Carson N.; Frei A.; Frey N. C.; Friederich P.; Gaudin T.; Gayle A. A.; Jablonka K. M.; Lameiro R. F.; Lemm D.; Lo A.; Moosavi S. M.; Nápoles-Duarte J. M.; Nigam A.; Pollice R.; Rajan K.; Schatzschneider U.; Schwaller P.; et al. SELFIES and the future of molecular string representations. Patterns 2022, 3, 100588 10.1016/j.patter.2022.100588. 36277819 PMC9583042

[ref35] Gimadiev T. R.; Lin A.; Afonina V. A.; Batyrshin D.; Nugmanov R. I.; Akhmetshin T.; Sidorov P.; Duybankova N.; Verhoeven J.; Wegner J.; Ceulemans H.; Gedich A.; Madzhidov T. I.; Varnek A. Reaction Data Curation I: Chemical Structures and Transformations Standardization. Mol. Inf. 2021, 40, 2100119 10.1002/minf.202100119. 34427989

[ref36] Ahneman D. T.; Estrada J. G.; Lin S.; Dreher S. D.; Doyle A. G. Predicting reaction performance in C-N cross-coupling using machine learning. Science 2018, 360, 186–190. 10.1126/science.aar5169. 29449509

[ref37] Kearnes S. M.; Maser M. R.; Wleklinski M.; Kast A.; Doyle A. G.; Dreher S. D.; Hawkins J. M.; Jensen K. F.; Coley C. W. The Open Reaction Database. J. Am. Chem. Soc. 2021, 143, 18820–18826. 10.1021/jacs.1c09820. 34727496

[ref38] Eyke N. S.; Koscher B. A.; Jensen K. F. Toward Machine Learning-Enhanced High-Throughput Experimentation. Trends Chem. 2021, 3, 120–132. 10.1016/j.trechm.2020.12.001.

[ref39] Huffman M. A.; Fryszkowska A.; Alvizo O.; Borra-Garske M.; Campos K. R.; Canada K. A.; Devine P. N.; Duan D.; Forstater J. H.; Grosser S. T.; Halsey H. M.; Hughes G. J.; Jo J.; Joyce L. A.; Kolev J. N.; Liang J.; Maloney K. M.; Mann B. F.; Marshall N. M.; McLaughlin M.; et al. Design of an in vitro biocatalytic cascade for the manufacture of islatravir. Science 2019, 366, 1255–1259. 10.1126/science.aay8484. 31806816

[ref40] Liu R. Copper-Catalyzed Enantioselective Hydroamination of Alkenes. Org. Synth. 2018, 95, 80–96. 10.15227/orgsyn.095.0080. 30287975 PMC6168011

[ref41] Christensen M.; Adedeji F.; Grosser S.; Zawatzky K.; Ji Y.; Liu J.; Jurica J. A.; Naber J. R.; Hein J. E. Development of an automated kinetic profiling system with online HPLC for reaction optimization. React. Chem. Eng. 2019, 4, 1555–1558. 10.1039/C9RE00086K.

[ref42] Zuo Z.; Ahneman D. T.; Chu L.; Terrett J. A.; Doyle A. G.; MacMillan D. W. C. Merging photoredox with nickel catalysis: Coupling of *α*-carboxyl sp sup3/sup -carbons with aryl halides. Science 2014, 345, 437–440. 10.1126/science.1255525. 24903563 PMC4296524

[ref43] Gioiello A.; Rosatelli E.; Teofrasti M.; Filipponi P.; Pellicciari R. Building a Sulfonamide Library by Eco-Friendly Flow Synthesis. ACS Comb. Sci. 2013, 15, 235–239. 10.1021/co400012m. 23514257

[ref44] Stadler A.; Kappe C. O. Automated Library Generation Using Sequential Microwave-Assisted Chemistry. Application toward the Biginelli Multicomponent Condensation. J. Comb. Chem. 2001, 3, 624–630. 10.1021/cc010044j. 11703160

[ref45] Nielsen M. K.; Ahneman D. T.; Riera O.; Doyle A. G. Deoxyfluorination with Sulfonyl Fluorides: Navigating Reaction Space with Machine Learning. J. Am. Chem. Soc. 2018, 140, 5004–5008. 10.1021/jacs.8b01523. 29584953

[ref46] Kutchukian P. S.; Dropinski J. F.; Dykstra K. D.; Li B.; DiRocco D. A.; Streckfuss E. C.; Campeau L.-C.; Cernak T.; Vachal P.; Davies I. W.; Krska S. W.; Dreher S. D. Chemistry informer libraries: a chemoinformatics enabled approach to evaluate and advance synthetic methods. Chem. Sci. 2016, 7, 2604–2613. 10.1039/C5SC04751J. 28660032 PMC5477042

[ref47] Schwärzer K.; Rout S. K.; Bessinger D.; Lima F.; Brocklehurst C. E.; Karaghiosoff K.; Bein T.; Knochel P. Selective functionalization of the 1iH/i-imidazo[1, 2-ib/i]pyrazole scaffold. A new potential non-classical isostere of indole and a precursor of push–pull dyes. Chem. Sci. 2021, 12, 12993–13000. 10.1039/D1SC04155J. 34745530 PMC8513920

[ref48] Newman-Stonebraker S.; Smith S.; Borowski J.; Peters E.; Gensch T.; Johnson H.; Sigman M.; Doyle A. Linking Mechanistic Analysis of Catalytic Reactivity Cliffs to Ligand Classification. ChemRxiv 2021, 10.26434/chemrxiv.14388557.v1.

[ref49] Saebi M.; Nan B.; Herr J. E.; Wahlers J.; Guo Z.; Zurański A. M.; Kogej T.; Norrby P.-O.; Doyle A. G.; Chawla N. V.; Wiest O. On the use of real-world datasets for reaction yield prediction. Chem. Sci. 2023, 14, 4997–5005. 10.1039/D2SC06041H. 37206399 PMC10189898

[ref50] Mdluli V.; Diluzio S.; Lewis J.; Kowalewski J. F.; Connell T. U.; Yaron D.; Kowalewski T.; Bernhard S. High-throughput Synthesis and Screening of Iridium(III) Photocatalysts for the Fast and Chemoselective Dehalogenation of Aryl Bromides. ACS Catal. 2020, 10, 6977–6987. 10.1021/acscatal.0c02247.

[ref51] Dreher S. D.; Krska S. W. Chemistry Informer Libraries: Conception, Early Experience, and Role in the Future of Cheminformatics. Acc. Chem. Res. 2021, 54, 1586–1596. 10.1021/acs.accounts.0c00760. 33723992

[ref52] Schleinitz J.; Langevin M.; Smail Y.; Wehnert B.; Grimaud L.; Vuilleumier R. Machine Learning Yield Prediction from NiCOlit, a Small-Size Literature Data Set of Nickel Catalyzed C–O Couplings. J. Am. Chem. Soc. 2022, 144, 14722–14730. 10.1021/jacs.2c05302. 35939717

[ref53] Reaxys. https://www.reaxys.com/, accessed on 2022-02-08.

[ref54] NextMove. https://nextmovesoftware.com, accessed on 2022-03-07.

[ref55] Fitzner M.; Wuitschik G.; Koller R. J.; Adam J.-M.; Schindler T.; Reymond J.-L. What can reaction databases teach us about Buchwald–Hartwig cross-couplings?. Chem. Sci. 2020, 11, 13085–13093. 10.1039/D0SC04074F. 34476050 PMC8378852

[ref56] Strieth-Kalthoff F.; Sandfort F.; Kühnemund M.; Schäfer F. R.; Kuchen H.; Glorius F. Machine Learning for Chemical Reactivity: The Importance of Failed Experiments. Angew. Chem., Int. Ed. 2022, 61, 10.1002/anie.202204647. 35512117

[ref57] Maloney M. P.; Coley C. W.; Genheden S.; Carson N.; Helquist P.; Norrby P.-O.; Wiest O. Negative Data in Data Sets for Machine Learning Training. Org. Lett. 2023, 25, 2945–2947. 10.1021/acs.orglett.3c01282. 37126483

[ref58] Jablonka K. M.; Patiny L.; Smit B. Making the collective knowledge of chemistry open and machine actionable. Nat. Chem. 2022, 14, 365–376. 10.1038/s41557-022-00910-7. 35379967

[ref59] Mehr S. H. M.; Craven M.; Leonov A. I.; Keenan G.; Cronin L. A universal system for digitization and automatic execution of the chemical synthesis literature. Science 2020, 370, 101–108. 10.1126/science.abc2986. 33004517

[ref60] Qian Y.; Guo J.; Tu Z.; Coley C. W.; Barzilay R. RxnScribe: A Sequence Generation Model for Reaction Diagram Parsing. J. Chem. Inf. Model. 2023, 63, 4030 10.1021/acs.jcim.3c00439. 37368970

[ref61] Wilary D. M.; Cole J. M. ReactionDataExtractor: A Tool for Automated Extraction of Information from Chemical Reaction Schemes. J. Chem. Inf. Model. 2021, 61, 4962–4974. 10.1021/acs.jcim.1c01017. 34525303

[ref62] Morgan H. L. The Generation of a Unique Machine Description for Chemical Structures-A Technique Developed at Chemical Abstracts Service. J. Chem. Doc. 1965, 5, 107–113. 10.1021/c160017a018.

[ref63] Varnek A.; Fourches D.; Hoonakker F.; Solov’ev V. P. Substructural fragments: an universal language to encode reactions, molecular and supramolecular structures. J. Comput.-Aided Mol. Des. 2005, 19, 693–703. 10.1007/s10822-005-9008-0. 16292611

[ref64] Fujita S. Description of organic reactions based on imaginary transition structures. 1. Introduction of new concepts. J. Chem. Inf. Model. 1986, 26, 205–212. 10.1021/ci00052a009.

[ref65] Nugmanov R. I.; Mukhametgaleev R. N.; Akhmetshin T.; Gimadiev T. R.; Afonina V. A.; Madzhidov T. I.; Varnek A. CGRtools: Python Library for Molecule, Reaction, and Condensed Graph of Reaction Processing. J. Chem. Inf. Model. 2019, 59, 2516–2521. 10.1021/acs.jcim.9b00102. 31063394

[ref66] Schwaller P.; Hoover B.; Reymond J.-L.; Strobelt H.; Laino T. Extraction of organic chemistry grammar from unsupervised learning of chemical reactions. Sci. Adv. 2021, 7, 10.1126/sciadv.abe4166. PMC8026122 33827815

[ref67] Probst D.; Schwaller P.; Reymond J.-L. Reaction classification and yield prediction using the differential reaction fingerprint DRFP. Digital Discovery 2022, 1, 91–97. 10.1039/D1DD00006C. 35515081 PMC8996827

[ref68] Yang K.; Swanson K.; Jin W.; Coley C.; Eiden P.; Gao H.; Guzman-Perez A.; Hopper T.; Kelley B.; Mathea M.; Palmer A.; Settels V.; Jaakkola T.; Jensen K.; Barzilay R. Analyzing Learned Molecular Representations for Property Prediction. J. Chem. Inf. Model. 2019, 59, 3370–3388. 10.1021/acs.jcim.9b00237. 31361484 PMC6727618

[ref69] Heid E.; Green W. H. Machine Learning of Reaction Properties via Learned Representations of the Condensed Graph of Reaction. J. Chem. Inf. Model. 2022, 62, 2101–2110. 10.1021/acs.jcim.1c00975. 34734699 PMC9092344

[ref70] Rogers D.; Hahn M. Extended-Connectivity Fingerprints. J. Chem. Inf. Model. 2010, 50, 742–754. 10.1021/ci100050t. 20426451

[ref71] Xiong Z.; Wang D.; Liu X.; Zhong F.; Wan X.; Li X.; Li Z.; Luo X.; Chen K.; Jiang H.; Zheng M. Pushing the Boundaries of Molecular Representation for Drug Discovery with the Graph Attention Mechanism. J. Med. Chem. 2020, 63, 8749–8760. 10.1021/acs.jmedchem.9b00959. 31408336

[ref72] Lee S.; Park H.; Choi C.; Kim W.; Kim K. K.; Han Y.-K.; Kang J.; Kang C.-J.; Son Y. Multi-order graph attention network for water solubility prediction and interpretation. Sci. Rep. 2023, 13, 10.1038/s41598-022-25701-5. PMC9981901 36864064

[ref73] Chuang K. V.; Keiser M. J. Comment on “Predicting reaction performance in C–N cross-coupling using machine learning”. Science 2018, 362, 10.1126/science.aat8603. 30442776

[ref74] Zuranski A. M.; Martinez Alvarado J. I.; Shields B. J.; Doyle A. G. Predicting Reaction Yields via Supervised Learning. Acc. Chem. Res. 2021, 54, 1856–1865. 10.1021/acs.accounts.0c00770. 33788552

[ref75] Sandfort F.; Strieth-Kalthoff F.; Kühnemund M.; Beecks C.; Glorius F. A Structure-Based Platform for Predicting Chemical Reactivity. Chem 2020, 6, 1379–1390. 10.1016/j.chempr.2020.02.017.

[ref76] Dong J.; Peng L.; Yang X.; Zhang Z.; Zhang P. scpXGBoost-based/scp intelligence yield prediction and reaction factors analysis of amination reaction. J. Comput. Chem. 2022, 43, 289–302. 10.1002/jcc.26791. 34862652

[ref77] Viet Johansson S.; Gummesson Svensson H.; Bjerrum E.; Schliep A.; Haghir Chehreghani M.; Tyrchan C.; Engkvist O. Using Active Learning to Develop Machine Learning Models for Reaction Yield Prediction. Mol. Inf. 2022, 41, 2200043 10.1002/minf.202200043. 35732584

[ref78] Eyke N. S.; Green W. H.; Jensen K. F. Iterative experimental design based on active machine learning reduces the experimental burden associated with reaction screening. React. Chem. Eng. 2020, 5, 1963–1972. 10.1039/D0RE00232A.

[ref79] Chen K.; Chen G.; Li J.; Huang Y.; Wang E.; Hou T.; Heng P.-A. MetaRF: attention-based random forest for reaction yield prediction with a few trails. J. Cheminf. 2023, 15, 10.1186/s13321-023-00715-x. PMC10084704 37038222

[ref80] Haywood A. L.; Redshaw J.; Hanson-Heine M. W. D.; Taylor A.; Brown A.; Mason A. M.; Gärtner T.; Hirst J. D. Kernel Methods for Predicting Yields of Chemical Reactions. J. Chem. Inf. Model. 2022, 62, 2077–2092. 10.1021/acs.jcim.1c00699. 34699222

[ref81] Ranković B.; Griffiths R.-R.; Moss H. B.; Schwaller P. Bayesian optimization for additive screening and yield improvements in chemical reactions – beyond one-hot encoding. ChemRxiv 2023, 10.26434/chemrxiv-2022-nll2j-v3.

[ref82] Fitzner M.; Wuitschik G.; Koller R.; Adam J.-M.; Schindler T. Machine Learning C–N Couplings: Obstacles for a General-Purpose Reaction Yield Prediction. ACS Omega 2023, 8, 3017–3025. 10.1021/acsomega.2c05546. 36713686 PMC9878668

[ref83] Reker D.; Hoyt E. A.; Bernardes G. J.; Rodrigues T. Adaptive Optimization of Chemical Reactions with Minimal Experimental Information. Cell Rep. Phys. Sci. 2020, 1, 100247 10.1016/j.xcrp.2020.100247.

[ref84] Vaswani A.; Shazeer N.; Parmar N.; Uszkoreit J.; Jones L.; Gomez A. N.; Kaiser L. u.; Polosukhin I. Attention Is All You Need. In Advances in Neural Information Processing Systems*;* Guyon I., Luxburg U. V., Bengio S., Wallach H., Fergus R., Vishwanathan S., Garnett R., Eds.; Curran Associates, Inc., 2017; Vol. 30.

[ref85] Baraka S.; Kerdawy A. M. E. Multimodal Transformer-based Model for Buchwald-Hartwig and Suzuki-Miyaura Reaction Yield Prediction. arXiv 2022, 10.48550/ARXIV.2204.14062.

[ref86] Gilmer J.; Schoenholz S. S.; Riley P. F.; Vinyals O.; Dahl G. E. In Proceedings of the 34th International Conference on Machine Learning*;* Precup D., Ed.; PMLR, 2017; Vol. 70, pp 1263–1272.

[ref87] Sato A.; Miyao T.; Funatsu K. Prediction of Reaction Yield for Buchwald-Hartwig Cross-coupling Reactions Using Deep Learning. Mol. Inf. 2022, 41, 2100156 10.1002/minf.202100156. 34585854

[ref88] Jaeger S.; Fulle S.; Turk S. Mol2vec: Unsupervised Machine Learning Approach with Chemical Intuition. J. Chem. Inf. Model. 2018, 58, 27–35. 10.1021/acs.jcim.7b00616. 29268609

[ref89] Kwon Y.; Lee D.; Choi Y.-S.; Kang S. Uncertainty-aware prediction of chemical reaction yields with graph neural networks. J. Cheminf. 2022, 14, 10.1186/s13321-021-00579-z. PMC8750748 35012654

[ref90] Neves P.; McClure K.; Verhoeven J.; Dyubankova N.; Nugmanov R.; Gedich A.; Menon S.; Shi Z.; Wegner J. K. Global reactivity models are impactful in industrial synthesis applications. J. Cheminf. 2023, 15, 10.1186/s13321-023-00685-0. PMC9921076 36774523

[ref91] Yarish D.; Garkot S.; Grygorenko O. O.; Radchenko D. S.; Moroz Y. S.; Gurbych O. Advancing molecular graphs with descriptors for the prediction of chemical reaction yields. J. Comput. Chem. 2023, 44, 76–92. 10.1002/jcc.27016. 36264601

[ref92] Estrada J. G.; Ahneman D. T.; Sheridan R. P.; Dreher S. D.; Doyle A. G. Response to Comment on “Predicting reaction performance in C–N cross-coupling using machine learning”. Science 2018, 362, 10.1126/science.aat8763. 30442777

[ref93] Cortes C.; Vapnik V. Support-vector networks. Mach. Learn. 1995, 20, 273–297. 10.1007/BF00994018.

[ref94] Ho T. K. In Random decision forests Proceedings of 3rd international conference on document analysis and recognition*;* Montreal, QC, Canada, August 14–16, 1995; IEEE, 1995; Vol. 1, pp 278–282. 10.1109/ICDAR.1995.598994

[ref95] Friedman J. H. Greedy function approximation: a gradient boosting machine. Ann. Statist. 2001, 29, 1189–1232. 10.1214/aos/1013203451.

[ref96] Pedregosa F.; Varoquaux G.; Gramfort A.; Michel V.; Thirion B.; Grisel O.; Blondel M.; Prettenhofer P.; Weiss R.; Dubourg V.; Vanderplas J.; Passos A.; Cournapeau D.; Brucher M.; Perrot M.; Duchesnay E. Scikit-learn: Machine Learning in Python. J. Mach. Learn. Res. 2011, 12, 2825–2830.

