# Supplementary material for: When Yield Prediction Does Not Yield Prediction: An Overview of the Current Challenges
Source: J Chem Inf Model. 2023 Dec 20;64(1):42–56. doi: 10.1021/acs.jcim.3c01524 (PMC10778086; doi:10.1021/acs.jcim.3c01524)
Supplement: Supplementary file 1 — ci3c01524_si_001.pdf [file ci3c01524_si_001.pdf]

Supporting Information - When yield prediction does not yield prediction: an overview of the current challenges

Varvara Voinarovska,*,†,‡ Mikhail Kabeshov,† Dmytro Dudenko,¶ Samuel Genheden,† and Igor V. Tetko§

†*Molecular AI, Discovery Sciences, R&D, AstraZeneca, Gothenburg, Sweden*

‡*Technical University of Munich, Germany; TUM Graduate School, Faculty of Chemistry*

¶*Enamine Ltd., 78 Chervonotkatska str., 02094 Kyiv, Ukraine*

§*Institute of Structural Biology, Molecular Targets and Therapeutics Center, Helmholtz Munich – Deutsches Forschungszentrum für Gesundheit und Umwelt (GmbH), 85764 Neuherberg, Germany*

E-mail: varvara.voinarovska@az

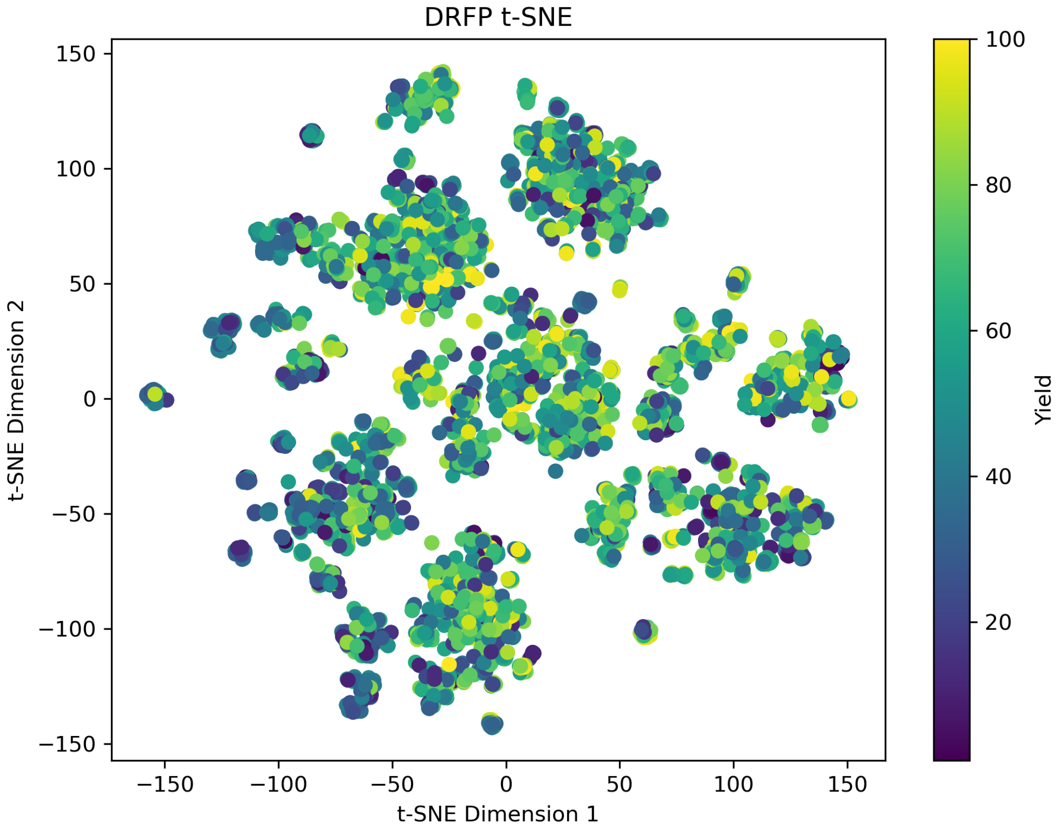

Figure 1: t-SNE for selected Buchwald-Hartwig amination from Reaxys.

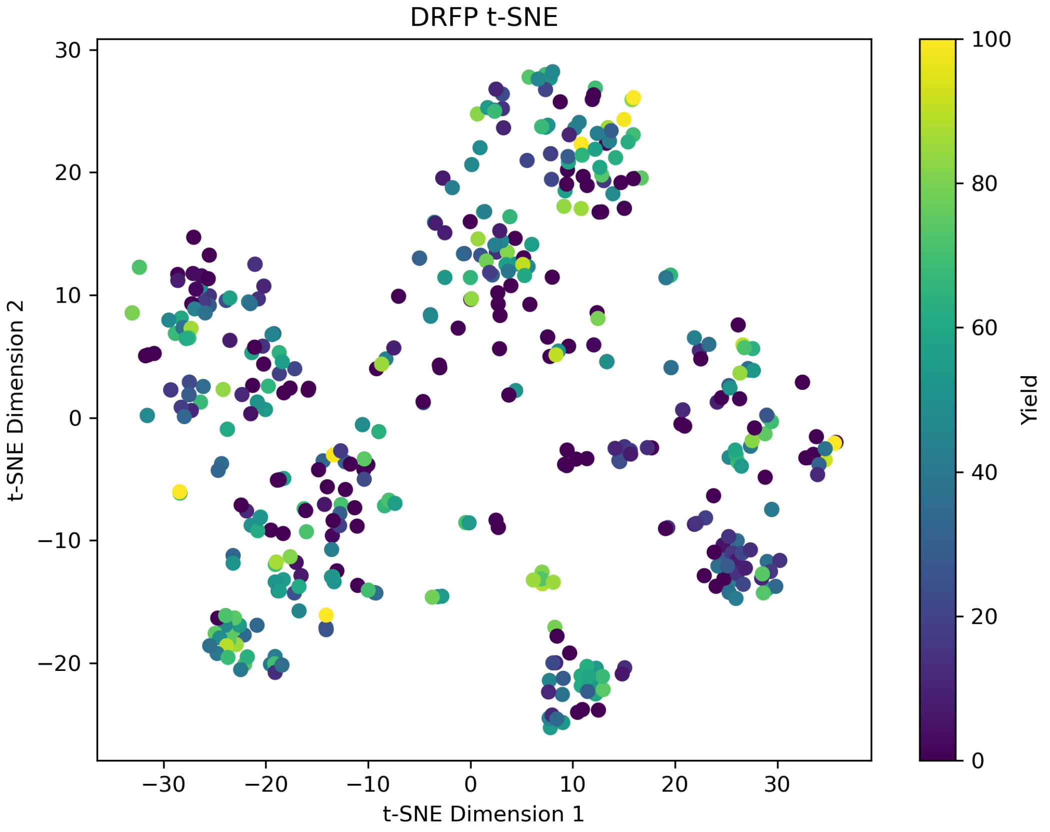

Figure 2: t-SNE for selected Buchwald-Hartwig amination from AZ ELN 750 dataset.

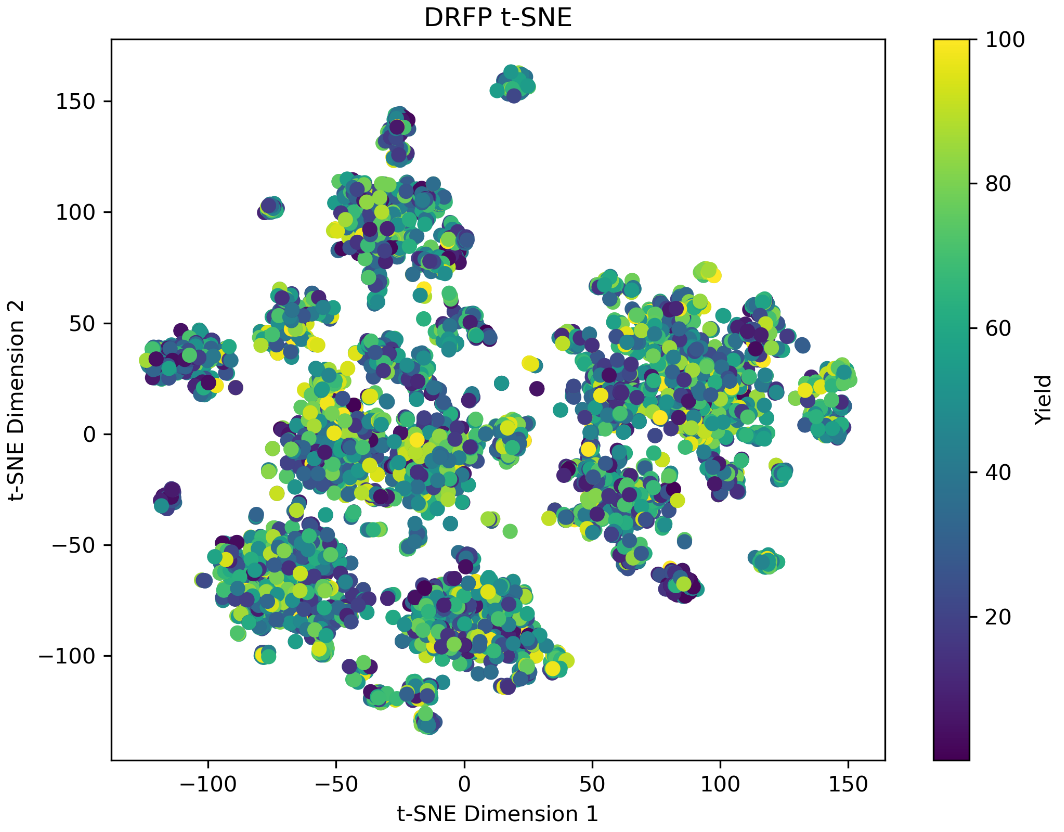

Figure 3: t-SNE for selected Buchwald-Hartwig amination from USPTO.

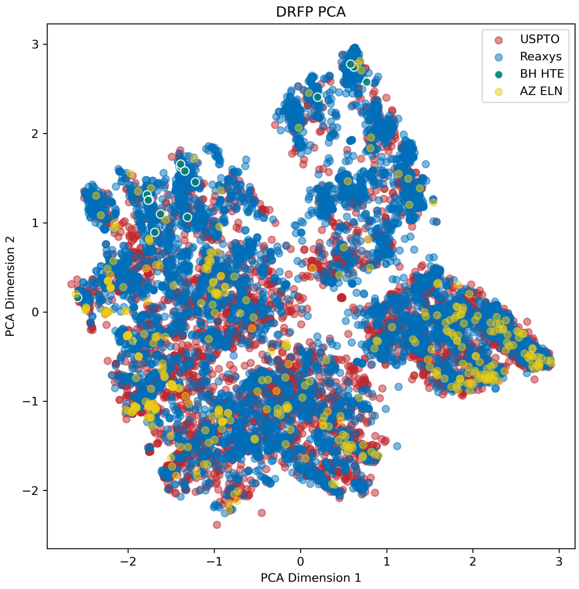

(a) Conditions excluded

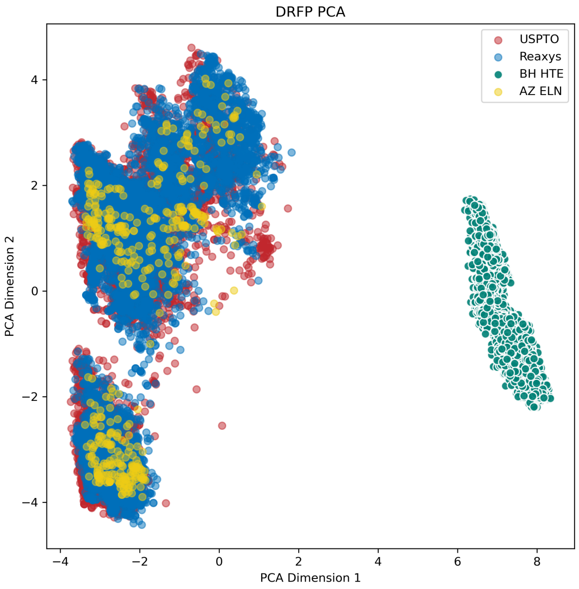

(b) Conditions included

Figure 4: The Principal Components Analysis plot depicts the distribution of reaction encodings based on DRFP representations. In 4a, where all conditions are excluded, the encodings show an even distribution in hyperspace. In 4b, when conditions are included, a notable separation occurs between the BH HTE dataset and others. This indicates that condition representations introduce diversity, adding a new layer of complexity to the encodings. We investigate the data recordings more in detail in the S11.

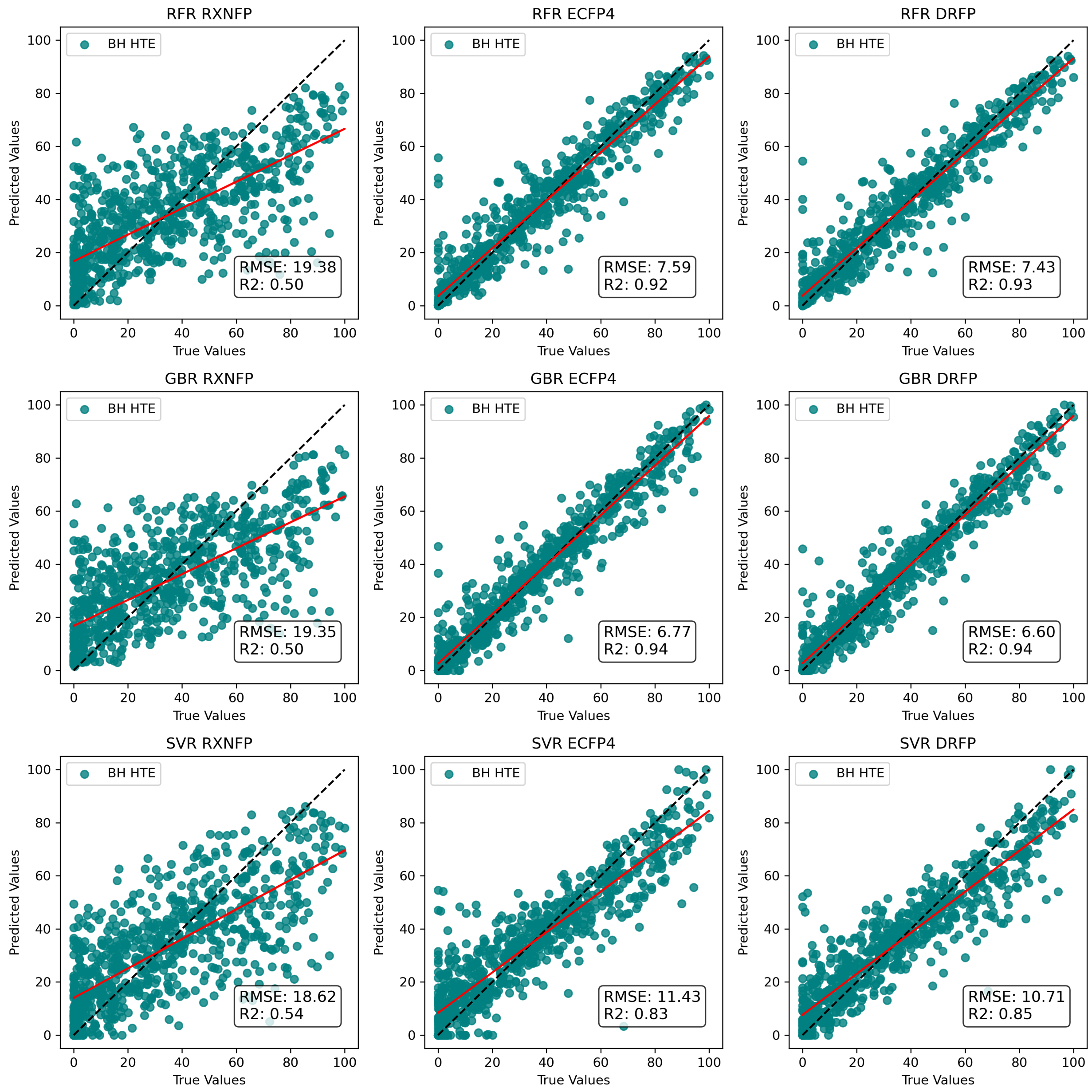

Figure 5: Comparison of the RFR, GBR, SVR model's performance using different encodings and fingerprints, trained with a random 80:20 ratio and 5-fold Cross-Validation.

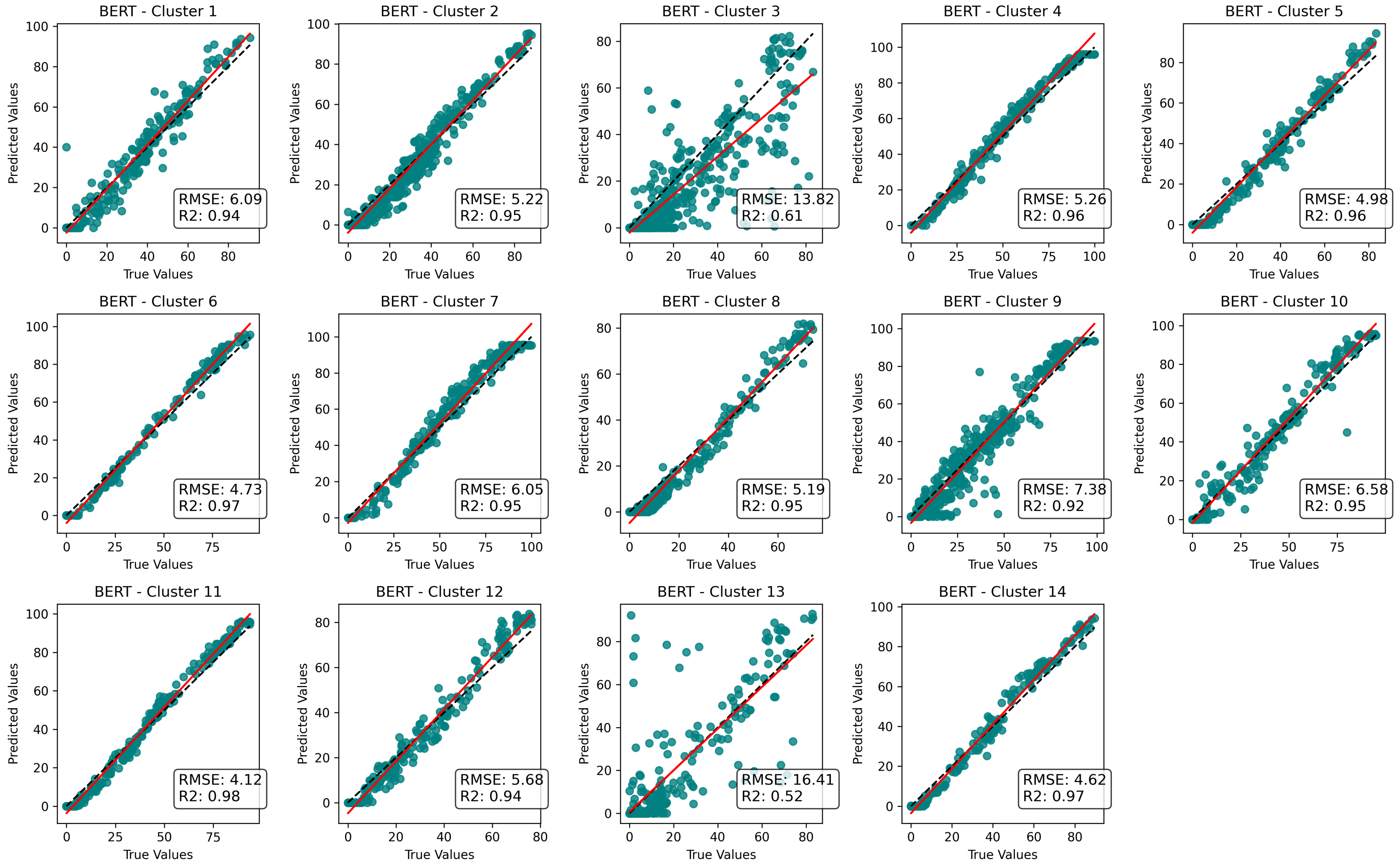

Figure 6: Yield-BERT performance on training one DRFP cluster left out

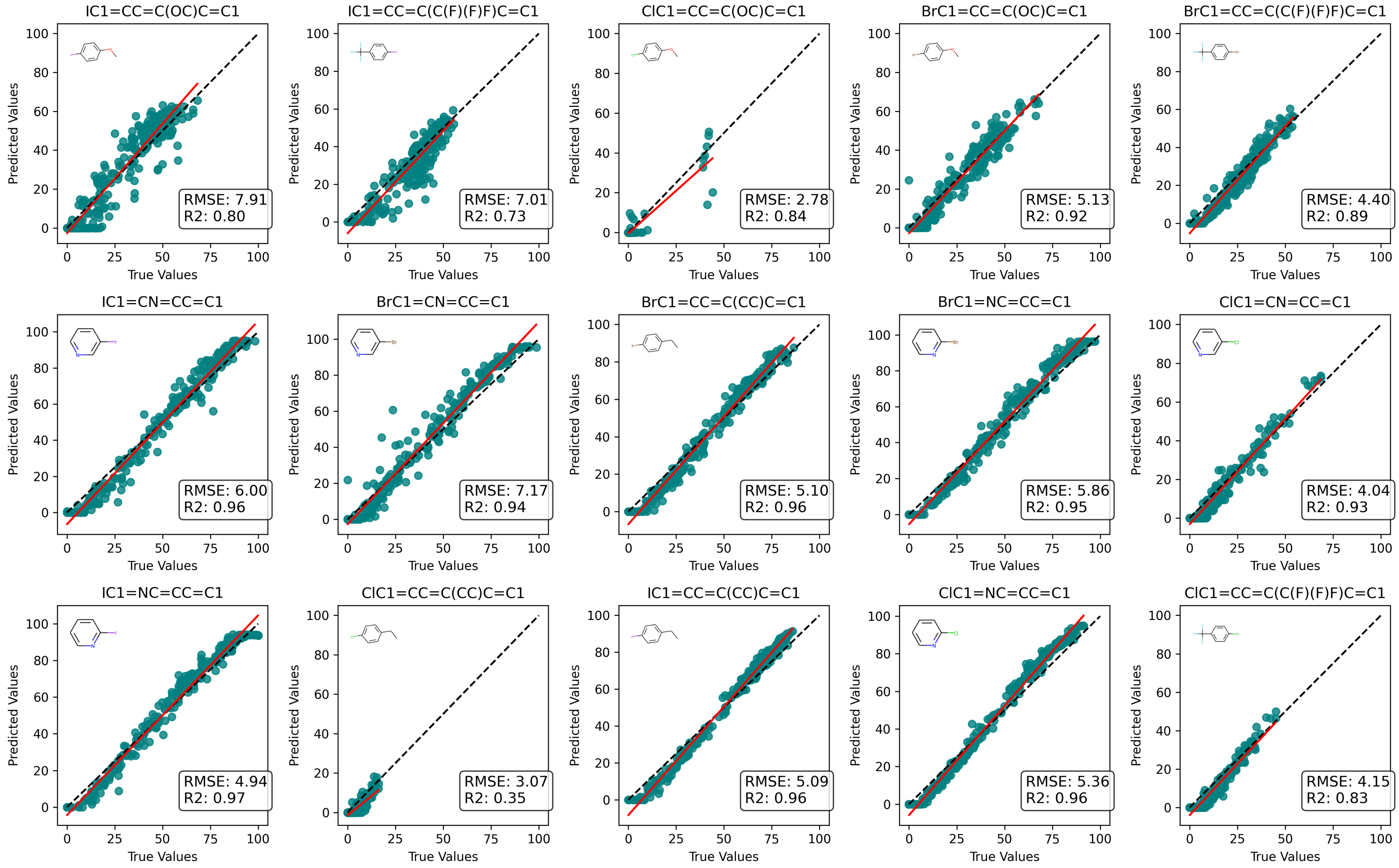

Figure 7: Yield-BERT performance on training one aryl halide cluster left out

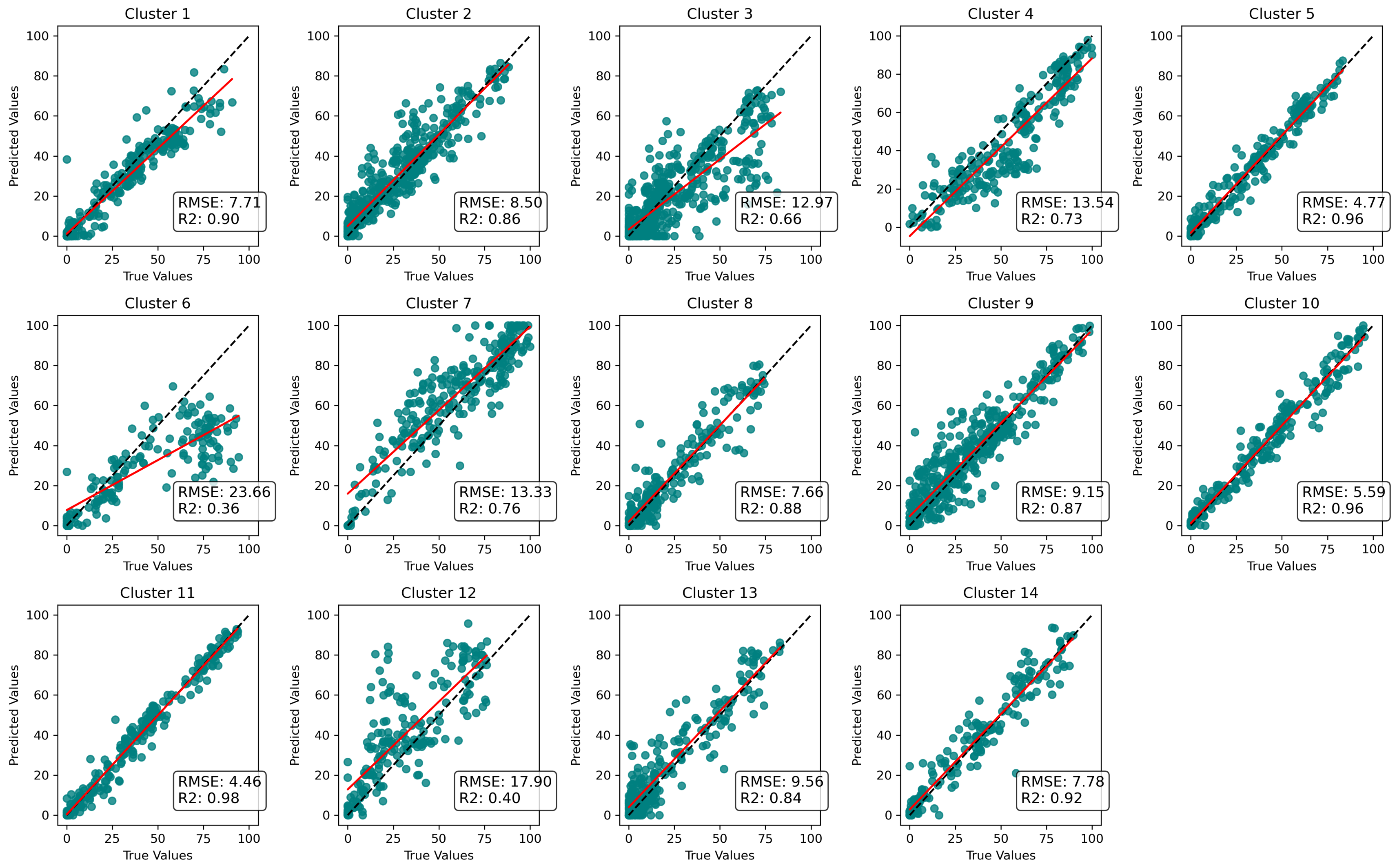

Figure 8: GBR performance on BH HTE DRFP features training one DRFP cluster left out

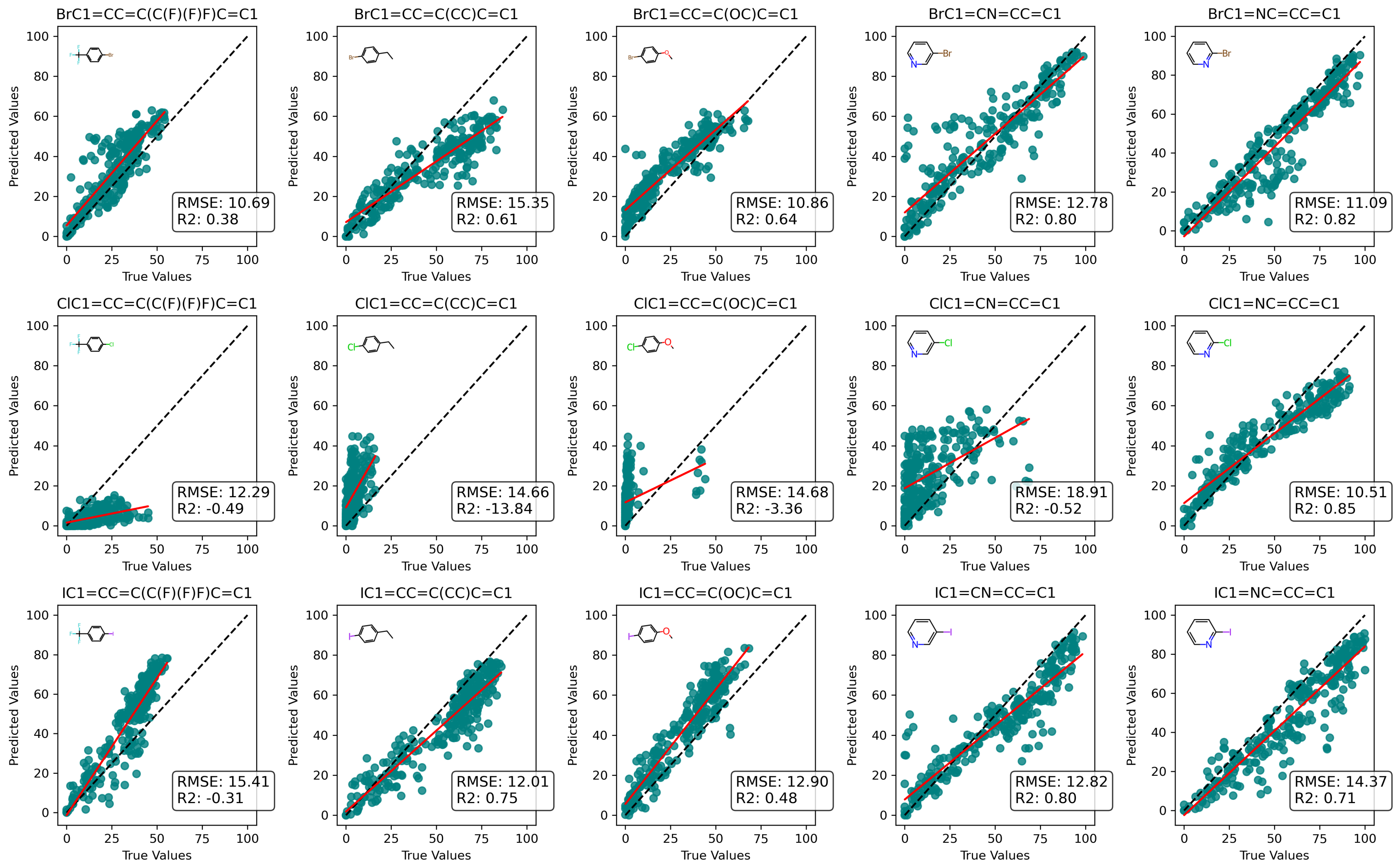

Figure 9: The performance of GBR on BH HTE DRFP features was evaluated by training the model with one aryl halide left out as a test set. Each plot represents the reactions of the left-out aryl halide used for testing.

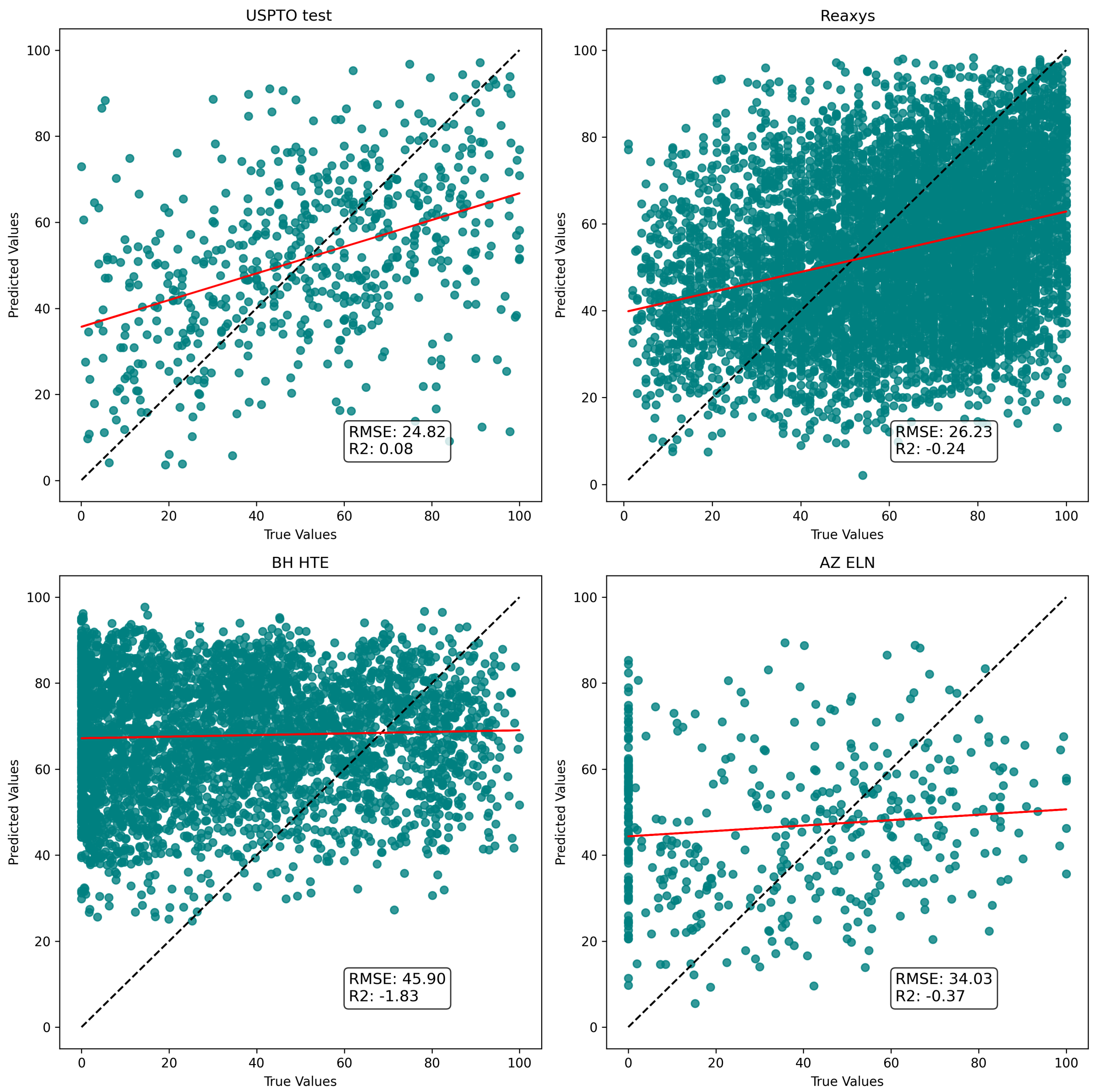

Figure 10: Yield-BERT model trained on USPTO Buchwald-Hartwig selection and tested on other datasets. The red line represents linear fit.

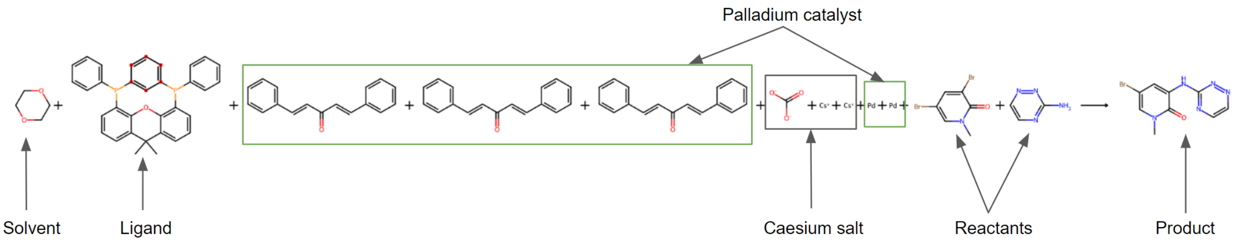

(a) USPTO ID01456115 example

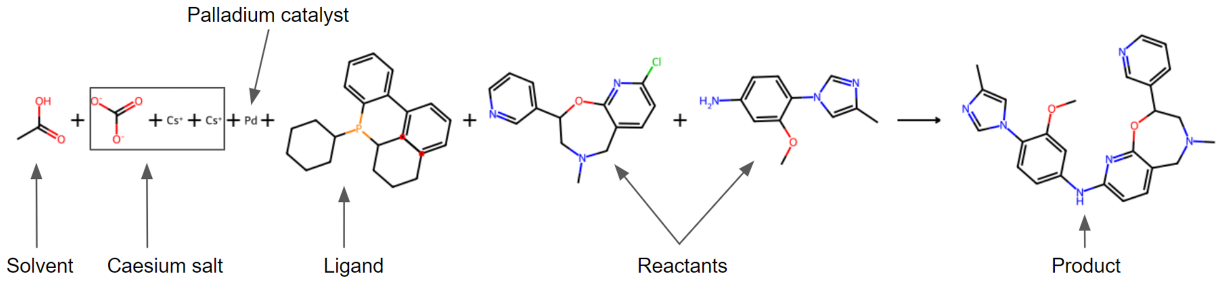

(b) AZ ELN example

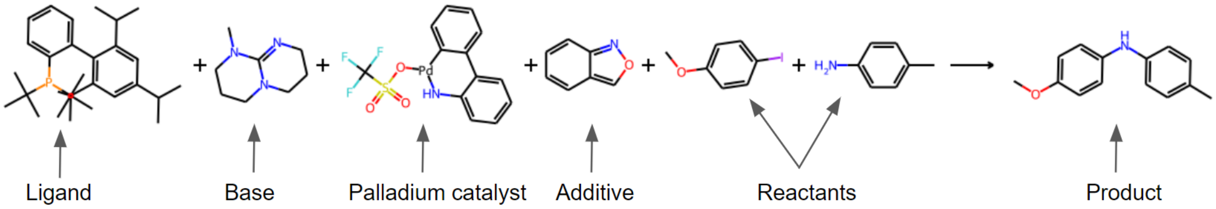

(c) BH HTE example

Figure 11: While essential components like aryl halides, amines, palladium catalysts, ligands, and bases are commonly used, variations in experimental conditions and the presence of additional additives or reaction components highlight the complexity of standardizing data for this reaction. This issue becomes particularly evident when comparing palladium catalyst representations across different data sources. For example, the catalyst appears "disassembled" in the USPTO entry, is represented as bare palladium in the AZ ELN, and takes the form of a complex pre-catalyst in the BH HTE dataset. These divergent encodings of the catalyst likely contribute to its separate clustering in t-SNE analysis.

Table 1: RMSE for the models employed in the research

			Test datasets RMSE			
Trained Model	Fingerprints	Training dataset	USPTO (test)	Reaxys (test)	BH HTE	AZ ELN
Random Forest Regression	RXNFP	USPTO	23.99	25.88	32.37	33.35
		Reaxys	28.54	20.70	40.29	40.26
	ECFP	USPTO	22.22	24.13	33.90	32.97
		Reaxys	26.72	18.25	43.30	39.15
	DRFP	USPTO	22.84	25.39	30.86	34.39
		Reaxys	28.31	18.70	42.26	40.23
Gradient Boost Regression	RXNFP	USPTO	24.29	26.17	31.86	33.63
		Reaxys	28.82	20.85	41.41	41.05
	ECFP	USPTO	22.39	24.18	34.75	32.03
		Reaxys	27.12	18.72	50.6	38.19
	DRFP	USPTO	23.41	25.93	32.47	33.28
		Reaxys	28.08	18.95	38.57	39.12
Support Vector Regression	RXNFP	USPTO	24.71	26.76	32.68	33.78
		Reaxys	30.53	21.72	42.27	43.82
	ECFP	USPTO	22.94	24.01	35.04	32.42
		Reaxys	27.60	18.9	48.29	40.76
	DRFP	USPTO	23.27	26.17	36.64	32.42
		Reaxys	28.98	19.56	48.75	41.37
BERT-Yield		USPTO	24.82	38.41	42.13	45.42
		Reaxys	38.45	20.99	47.18	48.61
